# The RNA Binding Protein ESRP1 Fine-Tunes the Expression of Pluripotency-Related Factors in Mouse Embryonic Stem Cells

**DOI:** 10.1371/journal.pone.0072300

**Published:** 2013-08-27

**Authors:** Sharmila Fagoonee, Claudia Bearzi, Ferdinando Di Cunto, John G. Clohessy, Roberto Rizzi, Markus Reschke, Emanuela Tolosano, Paolo Provero, Pier Paolo Pandolfi, Lorenzo Silengo, Fiorella Altruda

**Affiliations:** 1 Department of Molecular Biotechnology and Health Sciences, Molecular Biotechnology Center, University of Turin, Turin, Italy; 2 Multimedica IRCCS, Milan, Italy; 3 Institute of Cellular Biology and Neurobiology, National Council of Research, Rome, Italy; 4 Departments of Medicine and Pathology, Beth Israel Deaconess Medical Center, Harvard Medical School, Boston, Massachusetts, United States of America; National University of Singapore, Singapore

## Abstract

In pluripotent stem cells, there is increasing evidence for crosstalk between post-transcriptional and transcriptional networks, offering multifold steps at which pluripotency can be controlled. In addition to well-studied transcription factors, chromatin modifiers and miRNAs, RNA-binding proteins are emerging as fundamental players in pluripotency regulation. Here, we report a new role for the RNA-binding protein ESRP1 in the control of pluripotency. Knockdown of Esrp1 in mouse embryonic stem cells induces, other than the well-documented epithelial to mesenchymal-like state, also an increase in expression of the core transcription factors Oct4, Nanog and Sox2, thereby enhancing self-renewal of these cells. Esrp1-depleted embryonic stem cells displayed impaired early differentiation *in vitro* and formed larger teratomas *in vivo* when compared to control embryonic stem cells. We also show that ESRP1 binds to Oct4 and Sox2 mRNAs and decreases their polysomal loading. ESRP1 thus acts as a physiological regulator of the finely-tuned balance between self-renewal and commitment to a restricted developmental fate. Importantly, both mouse and human epithelial stem cells highly express ESRP1, pinpointing the importance of this RNA-binding protein in stem cell biology.

## Introduction

Pluripotency is a unique state in which cells can self-renew indefinitely whilst maintaining the ability to differentiate into multiple cell types of the body. In embryonic stem (ES) cells, gene regulatory networks comprising of the core transcription factors, Oct4, Nanog and Sox2 as well as chromatin regulatory proteins are involved in pluripotency maintenance [1]. High endogenous levels of these factors are beneficial for ES cell pluripotency, but deregulated expression of pluripotency-associated transcription factors has been shown to change cell fate. Small increases or decreases in Oct4 expression promote the differentiation of ES cells into extraembryonic endoderm and mesoderm or trophectoderm, respectively [2], [3]. Likewise, small increases in Sox2 can trigger the differentiation of ES cells into cells that express markers associated with a wide range of differentiated cell types [4]. Notably, there is increasing evidence for stemness and embryonic pathways reactivating during oncogenesis [5]. It is thus extremely important to deeper investigate the molecular mechanisms regulating the expression levels of the pluripotency factors.

The mechanisms that control the transcription of core pluripotency factors have been extensively studied [1], [3], [6], [7], [8]. In addition, several lines of evidence have recently underscored the importance of post-transcriptional regulation of gene expression in pluripotency maintenance [9]. To this regard, the action of microRNAs (miRNAs) as well as RNA-binding proteins (RBPs) involved in miRNA maturation, like dicer and dcgr8 has been investigated in detail [10], [11], [12], [13]. More recently, the RBP Unr (Upstream of N-*ras*) has been reported to post-transcriptionally repress Gata6 expression, causing stabilization of the pluripotent state of ES cells [14]. L1TD1, which interacts with Lin28, is also required for human ES cells self-renewal [15]. Despite this progress, the full contribution of RNA and of RBPs (RNA-based) to the complex regulatory circuitry of pluripotency is probably still underestimated, as suggested by the recent discovery that the expression of many large intergenic non-coding (lincRNAs) has effect on ES cell gene expression and on their differentiation state [16], [17]. In particular, the direct role of RBPs on the post-transcriptional regulation of core pluripotency factors expression in ES cells needs to be investigated in more depth. Genome-wide screenings aided by computational predictions may largely assist in this process [18].

To find new regulators of stemness/pluripotency, we analyzed conserved co-expression network (CCN) obtained from human and mouse stem-cell specific cDNA microarray data [19]. This analysis indicated the RBP Epithelial splicing regulatory protein 1 (Esrp1, also known as Rbm35a), first described as a tumor suppressor gene mutated in approximately 50% of primary colon tumors with microsatellite instability, as a protein possibly involved in pluripotency [20]. We found that depletion of ESRP1 in mouse ES cells resulted in increased self-renewal and impaired early differentiation *in vitro*. Moreover, ESRP1 binds to the mRNA of several pluripotency-related genes and decrease their polysomal loading, hence contributing to finely tune their expression levels in mouse ES cells. Altogether, our results indicate that ESRP1 is a new regulator of pluripotency.

## Materials and Methods

### Cell Culture and Differentiation

E14 ES cells were cultured and differentiated *in vitro* in embryoid bodies (EBs) as previously described [21], [22]. Briefly, 300 ES cells were cultured in EB differentiation media (see File S1 for details) in ultra-low attachment 96-well plates (Corning). Two days later, the EBs were collected and further cultured in ultra low attachment 6-cm dishes for the indicated times. Mouse spermatogonial stem cells (SSCs) were isolated from juvenile mice testis and cultured as previously described [23]. Epcam-positive SSCs were prepared from adult mice testis by MACS sorting [24] and cultured on inactivated Mefs as previously described [25]. See File S1 for details on SSC cultures.

### Generation of ES Cells with Stable Knockdown of ESRP1

Screening of short hairpin (Sh) RNA for efficient knockdown of Esrp1 in ES cells, vectors as well as lentiviruses production, quantitative real-time polymerase chain reaction (qRT-PCR), immunoblotting and immunofluorescence staining are described in File S1. Primers and probes employed for PCR and qRT-PCR are described in Table S1. Pluripotency, colony forming (alkaline phosphatase and methylene blue staining) and cell proliferation assays are also described in File S1. For rescue experiments, site directed mutagenesis was perfomed on pIBX-C-FF-EmGFP-B-ESRP1-2A (Kind gift of Pr. Russ Carstens) using QuikChange Site-Directed Mutagenesis Kit and following the manufacturer’s protocol (Stratagene). Primers used are described in Table S2. pIBX-C-FF-EmGFP (Kind gift of Pr. Russ Carstens) was used as control. Reverse transfection with lipofectamine 2000 was used for delivery plasmid DNA into ES cells (Invitrogen). See File S1 for further details.

### Teratoma Formation

Animals were bred in the central animal facility of the Molecular Biotechnology Center, University of Turin and were allowed free access to chow and drinking water and maintained under specific pathogen-free (SPF) conditions. Three hundred and fifty thousand Scramble (Scr) control or Esrp1-depleted ES cells were injected subcutaneously in recipient NOD-SCID-gamma (NSG) female mice. Teratomas were allowed to grow for 3 to 5 weeks. Mice were sacrificed using carbon dioxide euthanasia, followed by cervical dislocation to ameliorate suffering and teratomas were taken. Formalin- fixed, paraffin-embedded sections were stained with hematoxylin and eosin. For teratoma volume assessment, 5×10^4^ cells were injected as described above. Tumor volume was measured with a calliper, using the formula: ½ (length×width×height).

### Ethics Statement

All animal procedures were carried out under animal bioethics permit 116/92 (decreto legislativo 116/92) issued on the 11^th^ of September 2011 by the Bioethical committee of the University of Turin, Italy.

### RNA-immunuprecipitation

ES cells (Scr and Esrp1-depleted) were lysed and cytoplasmic extracts used for immunoprecipitation with anti-ESRP1 antibody or rabbit IgG and for RNA extraction as described in File S1.

### Sucrose Gradient Polysome Fractionation

ES cells (Scr and Esrp1-depleted) were incubated with cycloheximide (100 µg/ml, 15 min) and cytoplasmic lysates (200 µl) were fractionated by ultracentrifugation through 10–50% linear sucrose gradients and divided into 12 fractions for analysis. Where specified, the fractions were collected using an ISCO fractionator. RNA was extracted using Trizol and purelink RNA kit (Invitrogen) from pooled fractions 6–12 (polysomes). Protein was extracted using acetone/trichloroacetic acid precipitation. See File S1 for additional information.

### Western Blotting

Protein was extracted using TENT buffer (50 mM Tris-HCl, 5 mM EDTA, 150 mM NaCl, 1% Triton-X100) and a cocktail of protease inhibitors (Roche) and separated by SDS-PAGE. Fractionation of nuclear and cytoplasmic proteins is described in File S1. Antibodies used are described in Table S3. Cell culture and protein extraction for analysis of human ESRP1 expression in CD133+ kidney progenitor cells (KPC) [26] and kidney cancer stem cells (KCSC) [27] were generously provided by B. Bussolati and are described elsewhere. Densitometric analysis was performed using the volume analysis option of Quantity One software (Biorad Laboratories Inc).

### Reporter Assay

ES cells were transfected with firefly luciferase reporter plasmids (*Pou5f1*-luc [28], pGL3-CMV-SOX2-5′UTR [29] or Basic-luc control plasmid and control Renilla luciferase using Lipofectamine 2000 (Invitrogen). Forty-eight hours after transfection, cells were processed using a Dual-Luciferase Reporter Assay System (Promega) and reactions were read on a luminometer (Promega). Reporter firefly luciferase values were normalized to those of the control Renilla to evaluate transfection efficiencies.

### Statistical Analyses

Data are expressed as mean ± standard deviation. Statistical differences were determined by a 2-tailed Student’s *t*-test (*P<0.05, **P<0.01, ***P<0.001). All experiments were performed independently at least 3 times.

## Results

### Knockdown of Esrp1 Cells Results in Enhanced Self-renewal in Mouse ES Cells

To identify new RBPs that may affect self-renewal in pluripotent stem cells, we first selected a cluster of genes significantly down-regulated upon the onset of differentiation in mouse germline cell-derived pluripotent stem cells (GPSC), from our previously generated cDNA microarray data [22], [25]. Only those genes that display an expression profile strongly similar to key pluripotency factors in both human and mouse ES cells were selected among them, using a previously described stem-cell specific CCN [19]. As expected, the resulting short-list (Table S4) was strongly enriched for proteins well known to play a critical role in ES cell fate determination, such as SALL4, OCT4, DPPA4 and L1TD1 [1], [15], [30], [31]. Interestingly, this list also contained three RNA-binding proteins, the role of which has not been previously characterized in self-renewal of pluripotent cells: ELAV2, RBPMS2 and ESRP1. We decided to concentrate our functional validation on the latter, also considering that its expression has been strongly correlated to the expression of pluripotency-associated factors such as L1TD1, DNMT3B, LIN28 and TDGF1 by previous independent studies [15].

**Figure 1:**
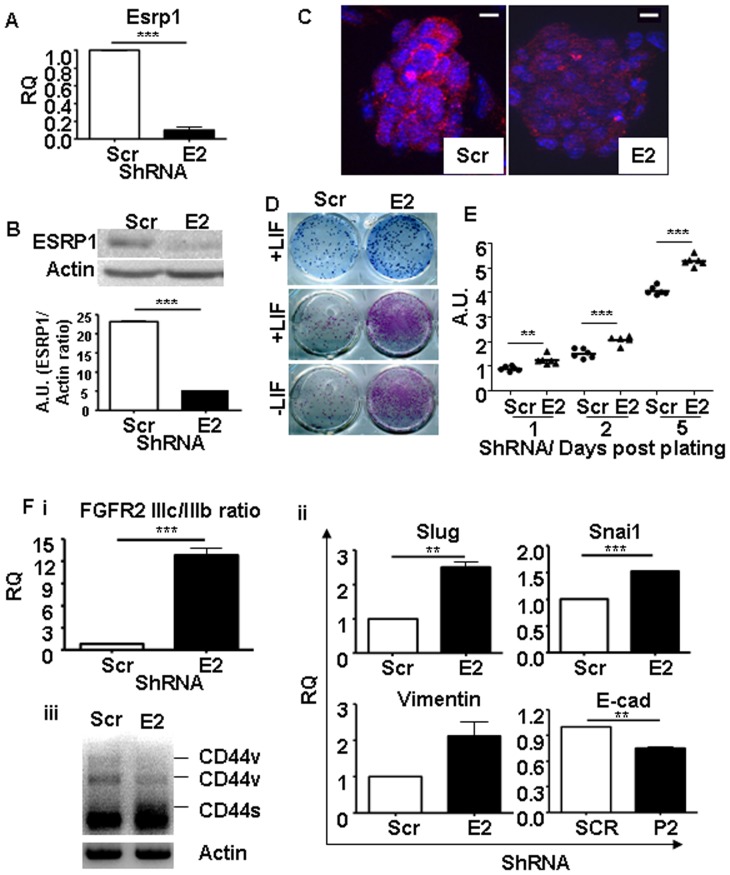
Knockdown of Esrp1 in E14 ES cells using short hairpin RNA. A. qRT-PCR analysis shows depletion of Esrp1 mRNA in Esrp1-depleted ES cells (E2) w.r.t. Scr controls; RQ is relative quantity (n = 3). B. Western blot analysis of Esrp1 expression in Scr and Esrp1-depleted ES cells. Densitometric analysis of the Western blot is shown; A.U. is arbitrary unit (n = 3). C. Immunofluorescence analysis of ESRP1 in Scr and Esrp1-depleted ES cells. Scale bar is 20 µm. D. Upper panel: Methylene blue staining of Scr and Esrp1-depleted ES cells plated on gelatin. Middle and lower panels: Alkaline phosphatase (ALP) staining of Scr and Esrp1-depleted ES cells in presence and absence of LIF, respectively, showing higher number of pluripotent colonies upon Esrp1 depletion. E. MTT assay perfomed on Scr and Esrp1-depleted ES cells at different time points shows higher proliferation rates of the latter (n = 6). F. qRT-PCR analysis shows an increase in (i) FGFR2 IIIc/IIIb ratio and (ii) Slug, Snai1 and Vimentin in Esrp1-depleted ES cells compared to Scr controls (n = 4). A slight decrease in E-cadherin (E-cad) was also observed. (iii) CD44 was amplified by PCR and shows a shift from variable isoform (CD44v) to standard isoform (CD44s) upon ESRP1 depletion indicating acquisition of a “mesenchymal” phenotype.

To examine the function of ESRP1 in mouse ES cells, we knocked down the endogenous ESRP1 expression. ShRNA- mediated depletion of ESRP1 by lentivirus in ES cells under basal conditions was confirmed both at the mRNA level, by qRT-PCR, and at the protein level, by western blotting and immunofluorescence analyses (Figure 1A–C).

The conserved coexpression of Esrp1 with the main pluripotency genes and its down-modulation during pluripotent cells differentiation suggested that it could be a positive regulator of pluripotency. However, in contrast with this prediction, methylene blue and alkaline phosphatase (ALP) staining showed that Esrp1-depleted ES cells formed undifferentiated colonies with greater efficiency than controls, both in the presence and in the absence of LIF. Scr ES cells differentiated in the absence of LIF and gave reduced ALP positivity. These results suggest that a reduction in ESRP1 may support LIF-independent ES cell propagation (Figure 1D). Accordingly, Esrp1-depleted cells proliferated at higher rate than Scr controls (Figure 1E). A second, independent ShRNA against Esrp1 gave similar results (Figure S1). On inactivated mouse embryonic fibroblasts (Mefs), Esrp1-depleted ES cell colonies were morphologically similar to controls and stained positive for OCT4 and NANOG (Figure S2).

As ESRP1 is well known for its involvement in maintaining an epithelial phenotype, we analysed the EMT status of the ES cells depleted for ESRP1 (Figure 1F). There was a statistically significant increase in the FGFR2 IIIc/IIIb ratio in these cells compared to Scr controls. This was accompanied by a switch from the CD44v to CD44s isoform upon knockdown of ESRP1, as well as an increase in Slug, Snai1 and Vimentin which are known markers of EMT. These changes were confirmed with another ShRNA against ESRP1 (Figure S1). On the other hand, there was a slight but significant decrease in E-cadherin expression in the ESRP1-depleted ES cells (Figure 1F).

**Figure 2:**
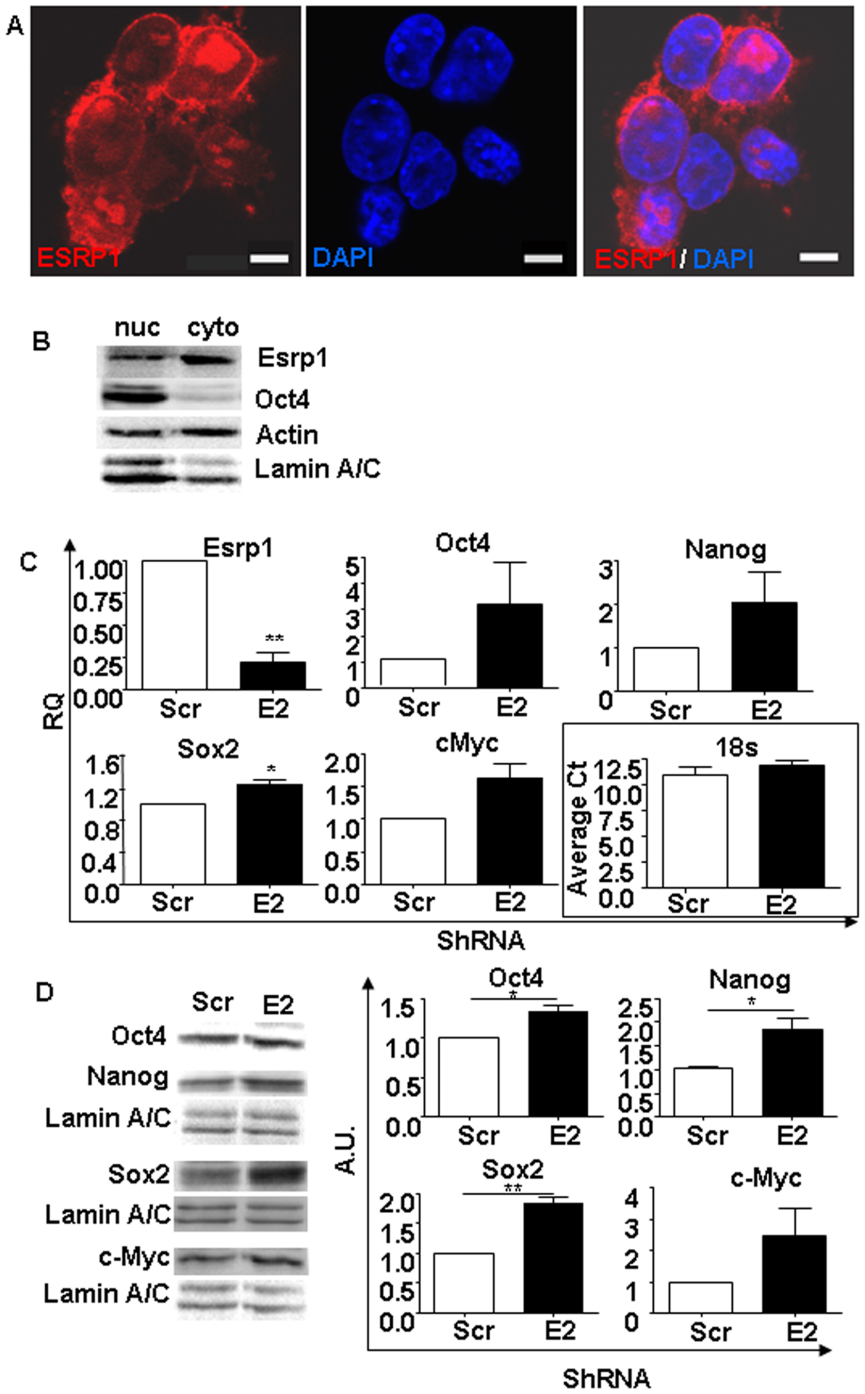
Pluripotency-related genes expression is affected by ESRP1 depletion. A. Confocal microscopy analysis of ESRP1 (red) in mouse ES cells reveal that the protein is expressed not only in the nucleus by also in the cytoplasm of these cells. Nuclei are stained with DAPI (blue). Scale bar is 5 µm. B. Representative Western blot analysis shows that ESRP1 is expressed mainly in the cytoplasm of mouse ES cells. As expected, Oct4 has a main nuclear localisation. Actin and Lamin A/C were used for normalisation. C. qRT-PCR analysis of Esrp1, Oct4, Nanog, Sox2, and c-Myc mRNA in Scr and Esrp1-depleted ES cells shows that there was a increase in their expression. RQ is relative quantity (n = 4). D. Representative Western blot analysis of Oct4, Nanog, Sox2 and c-Myc expression in nuclear extracts from Scr and Esrp1-depleted ES cells. Densitometric analysis of the Western blots is shown; A.U. is arbitrary unit (n = 3).

Confocal microscopy analysis of E14 ES cells revealed that ESRP1 was localized both in the nucleus and in the cytoplasm. Fractionation of nuclear and cytoplasmic proteins showed that ESRP1 was mainly located in the cytoplasm in the E14 ES cells (Figure 2A and B), in good agreement with previous reports on LS180 colon carcinoma cells [32]. Analysis of another ES cell line, v6.5, confirmed these results (Figure S3). This finding, together with the fact that ESRP1 was found to bind to c-Myc and regulate its expression, prompted us to investigate whether ESRP1 could be involved in the regulation of pluripotency- related genes as well. We thus measured the levels of the core pluripotency factors, Oct4, Nanog and Sox2 as well as the Esrp1 target c-Myc, (Figure 2C and D) and found that they were higher, albeit moderately, both at mRNA and protein levels with respect to Scr controls [32].

**Figure 3:**
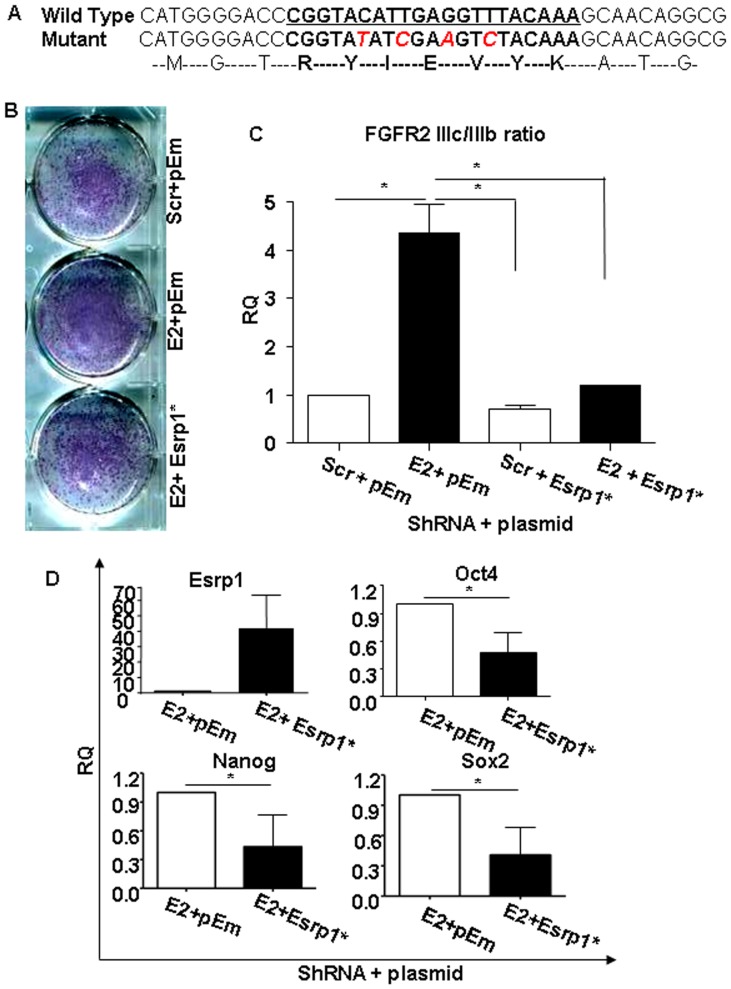
Rescue of Esrp1-depleted cells and differentiation potential of Esrp1-depleted ES cells. A. Sequence of Esrp1 cDNA showing ShRNA binding site (in bold and underlined) and the position of inserted mutations (red). The corresponding amino acid sequence is shown as well. B. Alkaline phosphatase (ALP) staining of Scr and Esrp1-depleted ES cells transiently transduced either with empty vector (pEm) and with the ShRNA-immune Esrp1-GFP cDNA (Esrp1*) (n = 3). C. qRT-PCR analysis of FGFR2 IIIc/IIIb ratio in Scr and Esrp1-depleted ES cells transiently transduced either with empty vector (pEm) and with the ShRNA-immune Esrp1-GFP cDNA (Esrp1*) showing a reduction in this ratio upon rescue. RQ is relative quantity (n = 3). D. qRT-PCR analysis of Esrp1, Oct4, Nanog and Sox2 mRNA in Esrp1-depleted ES cells transfected with pEm or Esrp1* expression vectors showing reduction in the expression of these genes upon rescue. RQ is relative quantity (n = 3).

In order to exclude the possibility of off-target effects of the ShRNAs, rescue experiments were performed using a mutant Esrp1. The four mutations inserted in the ShRNA binding site in pIBX-C-FF-EmGFP-B-ESRP1-2A plasmid (Figure 3A) did not alter ESRP1 expression when compared to a plasmid expressing the wild-type ESRP1 (Figure S4A) [33]. Importantly, transient introduction of this mutant plasmid into Esrp1-depleted ES cells reverted the phenotype observed after Esrp1 depletion. In particular, compared to cells transfected with the empty vector only, less ALP- positive colonies formed from Esrp1-depleted ES cells transfected with the ShRNA-immune Esrp1 (Figures 3B). Restoration of Esrp1 expression also induced a decrease in FGFR2 IIIc/IIIb ratio and in core pluripotency genes expression (Figure 3C and D, respectively) to a level that was comparable to that of Scr ES cells. Similar results were obtained with ES cells in a different genetic background (v6.5) (Figure S4B) or after knockdown with a second ShRNA against Esrp1 (Figure S4C). Taken together, these results indicate that Esrp1 was acting on self-renewal by regulating the expression of pluripotency transcription factors.

### Depletion of Esrp1 Enhances Induced Pluripotent Stem (iPS) Cell Colony Generation

To establish whether, besides to its role in ES cell proliferation and self renewal, ESRP1 may also play a role in pluripotency acquisition, its expression was analysed during the reprogramming of primary Mefs into iPS cells as previously described [34]. In accordance with previous studies, the expression of Esrp1 shows a trend similar to the core pluripotency factors starting at Day 8 of reprogramming and increasing progressively (Figure S5A) [35]. Moreover, depletion of Esrp1 in Mefs before the reprogramming procedure resulted in increased number of iPS cell colonies as evidenced by CDy1 and OCT4 staining compared to Scr controls or controls not infected with ShRNA-harbouring lentiviruses (NT) in agreement with the data on ES cells (Figure S5B and C, respectively) [36]. EBs generated from iPS resulting from the three conditions expressed markers of the three germ layers upon EBs formation (Figure S6A) and formed teratomas when injected into immuno-compromised mice (Figure S6B).

### Downregulation of Esrp1 in ES Cells Results in Impaired Early Differentiation Capacity

**Figure 4:**
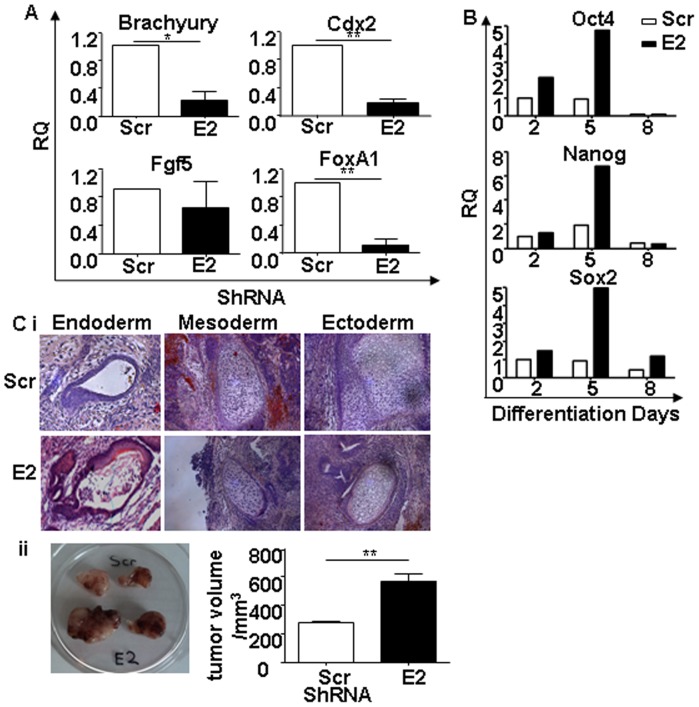
Analysis of differentiative potential of Esrp1-depleted ES cells. A. qRT-PCR analysis of Brachyury (mesoderm), Cdx2 (trophectoderm), Fgf5 (ectoderm) and FoxA1 (endoderm) mRNA in Scr and Esrp1-depleted embryoid bodies (EB) five days after the differentiation process. RQ is relative quantity (n = 4). B. Representative qRT-PCR analysis of Oct4, Nanog and Sox2 mRNA in Scr and Esrp1-depleted EBs at different time points. C i. Teratoma formation 4 weeks after subcutaneous injection of 350 000 Scr or Esrp1-depleted ES cells in NSG mice. Hematoxylin/eosin staining demonstrates the presence of all three germ layers (n = 8). ii. Teratomas generated at 19 days after subcutaneous injection of 50 000 Scr and Esrp1-depleted ES cells in NSG mice. Graph shows tumor volume measured with a calliper (n = 3).

To assess the impact of Esrp1 depletion on the differentiation capacity of ES cells, we generated EBs from Scr control and Esrp1-depleted ES cells. At Day 5 of differentiation *in vitro*, Esrp1-depleted ES cells showed impaired early differentiation, as indicated by the reduced levels of mesoderm (Brachyury) and endoderm (FoxA1) markers (Figure 4A). Ectoderm (Fgf5) marker expression was comparable (Figure 4A). In order to exclude the possibility that a reduction of ESRP1 expression might lead to trophectoderm differentiation of ES cells (as previously observed upon Oct4 and Sall4 depletion in ES cells [2], [30]), we also analysed the expression of trophectoderm (Cdx2) marker. A reduction in Cdx2 expression was observed in Esrp1-depleted ES cells compared to Scr ES cells (Figure 4A). Moreover, in the EBs derived from Esrp1-depleted ES cells, the expression of pluripotency-related genes remained elevated till Day 5 of differentiation and then become comparable to Scr controls by Day 8 (Figure 4B). In order to test whether the delay in differentiation was temporal or absolute, *in vivo* teratoma assay was performed by injecting 3.5×10^5^ Esrp1-depleted cells in NOD-SCID-Gamma (NSG) mice [37]. Resulting teratomas in 8 out of 8 mice per group comprised all the three germ layers showing that these cells are indeed pluripotent and that their differentiation defect is only temporal (Figure 4C). These data establish that the depletion of Esrp1 does not impair the multilineage differentiation potential of ES cells on the long term. Interestingly, the same assay, performed under non-saturating conditions (5×10^4^ ES cells) showed that Esrp1 KD ES cell-derived teratomas grew significantly faster that those derived from Scr control (Figure 4C). This was due to the higher proliferation rate, as evidenced by PCNA staining, of the more prominent neuroepithelium (asterisks and insets in Figure S7) in teratomas generated from Esrp1-depleted ES cells compared to Scr ES cells (Figure S7).

### ESRP1 is Expressed in Epithelial Stem Cells

**Figure 5:**
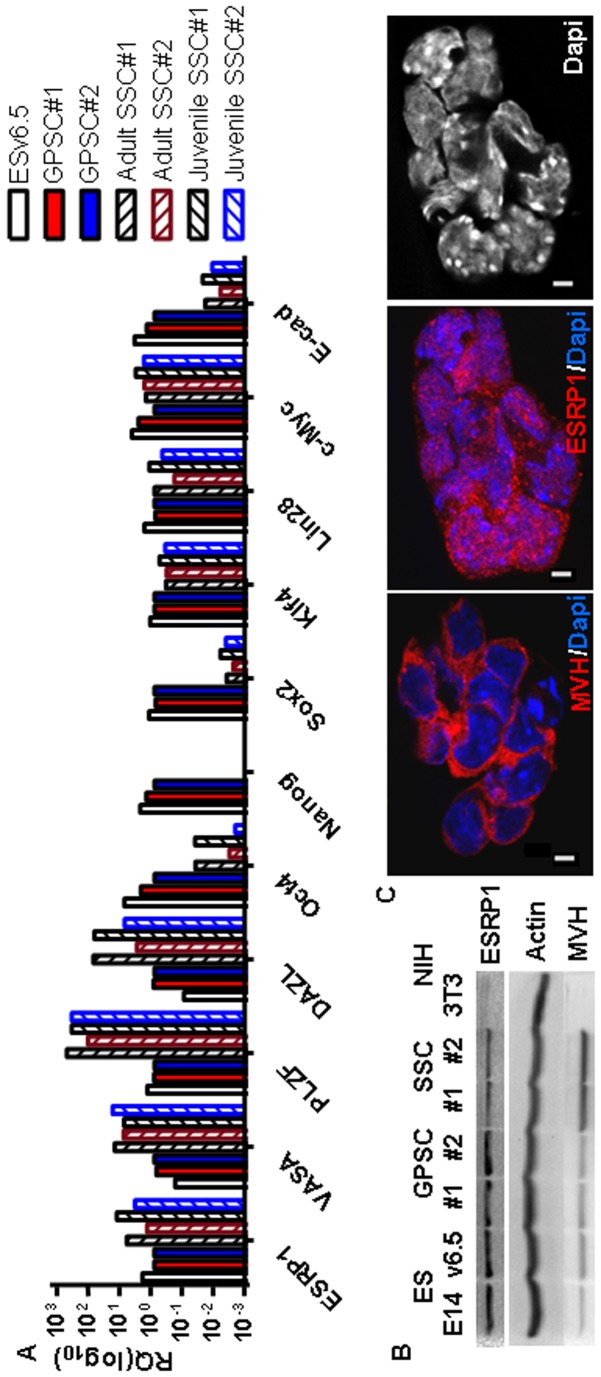
ESRP1 is expressed in spermatogonial stem cells. A. Spermatogonial stem cells (SSCs) were characterised by qRT-PCR analysis. RQ is relative quantity (n = 4). B. Western blot analysis shows that ESRP1 is expressed in ES cells, in GPSCs as well as in SSCs. ES cells and GPSCs were lysed after 30 minutes’ preplating to remove Mefs, while SSCs were scraped off together with the Mef-feeder layer for lysis. The primordial germ cell marker, MVH was enriched in SSCs versus the other cell lines. Actin was used for normalisation (n = 2). 3T3 fibroblasts were used as negative control. C. Confocal microcroscopy analysis revealed that ESRP1 is both nuclear and cytoplasmic in the SSCs as well. MVH staining was used as positive control. Scale bar is 5 µm.

In order to assess the applicability of these findings to other types of stem cells, we analysed the expression of ESRP1 in the unipotent spermatogonial stem cells or SSCs. As expected, the mouse SSCs express pluripotency markers like Oct4, Sox2, Klf4, Lin28, c-Myc as well as e-cadherin, but not Nanog compared to the pluripotent stem cells, ES and GPSCs [38], [39]. ESRP1 is expressed both at the RNA and protein levels in SSCs (Figure 5A and B). Interestingly, ESRP1 is present both in the nucleus and the cytoplasm of these cells, in accordance with the results obtained on ES cells (Figure 5C and Figure 2A, respectively). Moreover, ESRP1 is also highly expressed in CD133+ human kidney progenitor cells, but downregulated in kidney cancer stem cells (protein extracts kindly provided by B. Bussolati) (Figure S8) [26], [27].

### ESRP1 Binds to Pluripotency-related mRNAs

**Figure 6:**
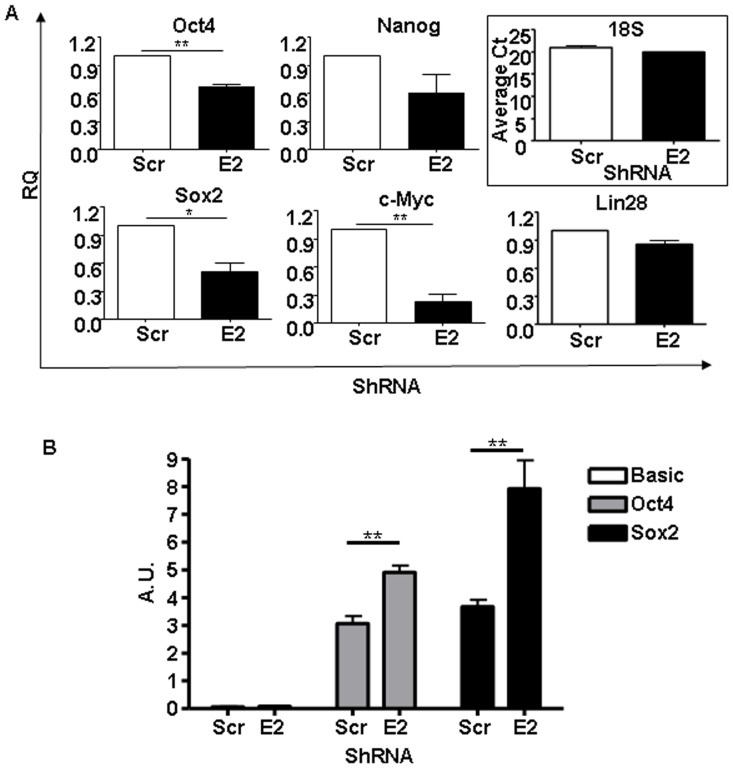
ESRP1 binds to pluripotency-related mRNAs. A. RNA-immunoprecipitation (RIP) was performed using an anti-ESRP1 antibody. qRT-PCR was employed to analyse for the presence of Oct4, Nanog, Sox2, c-Myc and Lin28 mRNA bound by ESRP1. ESRP1-depleted samples were used as negative control; RQ is relative quantity (n = 3). 18s mRNA was equally immunoprecipitated in both cell types and average threshold cycle (Ct) is shown. B. 5′UTR reporter luciferase assay of Scr and ESRP1-depleted ES cells transfected with pGL3-pCMV-5′UTR-Oct4 and pGL3-pCMV-5′UTR-Sox2 versus Basic pGL3 vector; A.U. is arbitrary unit (n = 6).

As a protein containing RNA-recognition motifs, ESRP1 has been reported to associate with multiple mRNAs at their 5′UTRs in the cytoplasm and to control their turnover and/or translational regulation [32]. To further analyse if pluripotency-related mRNAs are also bound by ESRP1, we performed RNA-immunoprecipitation with an anti-ESRP1 antibody on cytoplasmic extracts from mouse control ES cells. In agreement with the mentioned study, c-Myc mRNA was specifically immunoprecipitated in this assay, as no binding to the preimmune IgG (Figure S9) or in Mefs (Table S5) used as negative controls was detected (Figure 6A). Most interestingly, Oct4 and Sox2 mRNAs were consistently co-immunoprecipitated with ESRP1(Figure S9). Accordingly, when ESRP1-depleted ES cells were used, there was statistically significant reduction in these pluripotency-related mRNAs, but not in Nanog or Lin28 mRNA, in the ESRP1 immunoprecipitation compared to the Scr controls (Figure 6A). We further investigated whether ESRP1 could regulate the expression of Oct4 and Sox2 mRNAs through their 5′UTR regions, by analysing translation of firefly luciferase reporter using pGL3-pCMV-5′UTR-Oct4 and pGL3-pCMV-5′UTR-Sox2 versus basic pGL3 vector. The luciferase reporter assays show that there was a statistically significant increase in luciferase activity when ESRP1 was depleted in ES cells compared to Scr controls (Figure 6B), confirming that ESRP1 could inhibit the expression of Oct4 and Sox2 by binding their 5′UTR regions.

### Depletion of ESRP1 does not Affect the Decay Rate but Stimulates the Polysomal Loading of Pluripotency-related mRNAs

Since RBPs can regulate mRNA turnover, we investigated whether ESRP1 can influence the stability of pluripotency-related mRNAs. Actinomycin D-treated Scr and Esrp1-depleted ES cells showed similar mRNA decay rates of Oct4, Nanog, Sox2 and c-Myc, while as expected, there was a significant difference the Esrp1 mRNA degradation rate in these two cell types (Figure S10).

Thus, at least in our hands, ESRP1 does not affect the analysed pluripotency-related mRNA stability in mouse ES cells.

**Figure 7:**
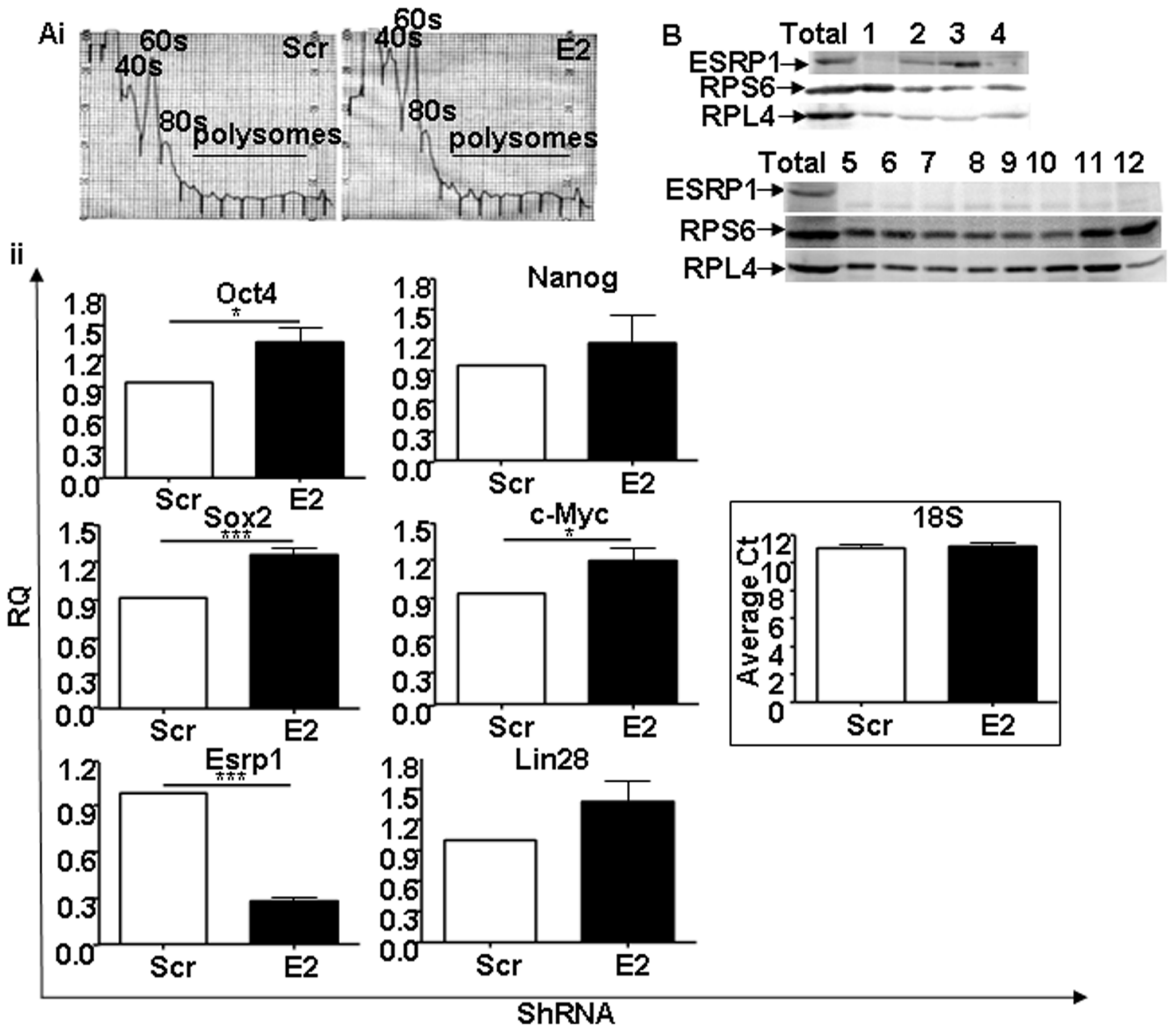
Polysomal loading of pluripotency-related mRNAs. A i. Polysome profiles of Scr and Esrp1-depleted ES cells obtained using an ISCO-fractionator. ii. qRT-PCR analysis shows the abundance of Oct4, Nanog, Sox2, c-Myc, Esrp1 and Lin28 mRNA in pooled polysomal fractions. 18s mRNA was equally immunoprecipitated in both cell types and average threshold cycle (Ct) is shown. RQ is relative quantity (n = 3). B. Representative Western blot analysis of ESRP1, RPS6 and RPL4 expression in the sucrose gradient fractions obtained from ES cells lysates under basal conditions.

It is generally believed that mRNAs actively being translated are associated with polysomes and that an increased polysome association indicates an increase in translation efficiency [40]. Based on this assumption, to provide further evidence supporting the inhibitory role of Esrp1 on mRNA translation, we performed polysomal profiling [32]. Cytoplasmic extracts from Scr and Esrp1-depleted ES cells were subjected to sucrose gradient fractionation and total RNA was extracted from polysomal fractions. While there was no difference in the ISCO fractionator-generated polysome profile of Scr and Esrp1-depleted ES cells (Figure 7A), the amount of core pluripotency-related factors and c-Myc mRNAs in the pooled polysomal fractions was significantly higher upon depletion of Esrp1 compared to controls (Figure 7A). These results suggest that the increased protein levels of pluripotency factors shown in Figure 2D are at least partially due to their increased translation. On the other hand, Nanog and Lin28 mRNA loading did not significantly differ between Scr and Esrp1-depleted ES cells (Figure 7A). To further investigate in which sucrose gradient fractions ESRP1 was found in ES cells under basal conditions, we extracted proteins from the different fractions. The results show that ESRP1 is not located in the polysomal fractions (Figure 7B), suggesting that ESRP1 may sequester pluripotency-related mRNAs away from the polysomes. Taken together, our findings indicate that depletion of ESRP1 in ES cells resulted in alteration in the level of expression of core transcription factors and the resultant cumulative effect of these changes affects early differentiation of these cells.

## Discussion

ESRP1 has been shown to affect the mRNA translation of several cancer-related genes, including c-Myc and Fos, through direct binding of their 5′ untranslated regions (UTRs) [20], [32]. Moreover, Warzecha et al. demonstrated that ESRP1 can promote the alternative splicing of transcripts that switch splicing during epithelial to mesenchymal transition (EMT) [33], [41], [42]. Its role in EMT and tumor progression is getting increasingly studied [43], [44], [45].

In this study, we identified the RBP ESRP1 as a candidate regulator of self renewal and pluripotency in ES cells on the basis of its conserved co-expression with well-established pluripotency factors. Moreover, and most importantly, we provide strong functional evidence in support of this hypothesis, showing that ESRP1 controls the translation of pluripotency-related mRNAs in mouse ES cells and that its downregulation favours reprogramming of differentiated fibroblasts into pluripotent cells.

The capability of ESRP1 to restrain self renewal could appear paradoxical if its expression profile is considered. Indeed, ESRP1 is downregulated with the other pluripotency factors when both mouse GPSC and ES cells lose pluripotency, and it is upregulated when fibroblasts are reprogrammed into iPS cells. However, the experiments that we performed clearly indicate that ESRP1 functionally counteracts the activity of pluripotency-related genes. Indeed, reduced levels of Esrp1 enhances ES cells proliferation and self renewal, as shown by the higher number of undifferentiated colonies generated *in vitro* and by the increased size of teratoma obtained upon injecting non-saturating amounts of cells into immune-compromised animals compared to Scr controls. In addition, Esrp1 knockdown increases the number of iPS cell colonies obtained from primary differentiated fibroblasts. An alternative explanation for these phenotypes could be that a reduction of Esrp1 could transform pluripotent cells, a conceivable scenario especially if considering the previous implication of human ESRP1 as a tumor suppressor gene and in EMT [32], [33]. However, our data seem to exclude this possibility. Indeed, Esrp1-depleted cells are still capable of responding to differentiative stimuli, albeit with a slower kinetic when compared to control cells. Moreover, teratoma formation assays showed that these cells are still capable of giving rise to derivatives of the three germ layers. On the basis of these data, it seems safe to us to conclude that, in pluripotent cells, ESRP1 is a physiological regulator of the finely tuned balance between self renewal and commitment to a restricted developmental fate.

The simplest explanation to reconcile the functional properties of Esrp1 with its expression profile could be that the same transcriptional program that sustains the expression of pluripotency factors is responsible for the activation of a negative control layer, composed of Esrp1, and possibly, by other coding or non-coding genes. The existence of such negative fine-tuning would be very helpful to explain how the pluripotency machinery is kept under strict control, if considering that it is maintained in pluripotent cells by strong positive feedback loops between the core transcription factors [46]. An important next step to further support this scenario will be to investigate how Esrp1 is regulated at the transcriptional level. Interestingly, CHIP-seq data indicated that its promoter contains binding sites for both polycomb repressive complex (PRC) members (PRC2: EZH2 and SUZ12 and PRC1: RING1B) as well as for pluripotency-related genes (NANOG, KLF4, TCF3), suggesting that ESRP1 level in mouse ES cells is controlled by both pluripotency factors as well as PRC members [46], [47]. However, further studies are needed to assess how important these factors are for the regulation of ESRP1 in ES cells under basal conditions and in response to differentiative stimuli.

**Figure 8:**
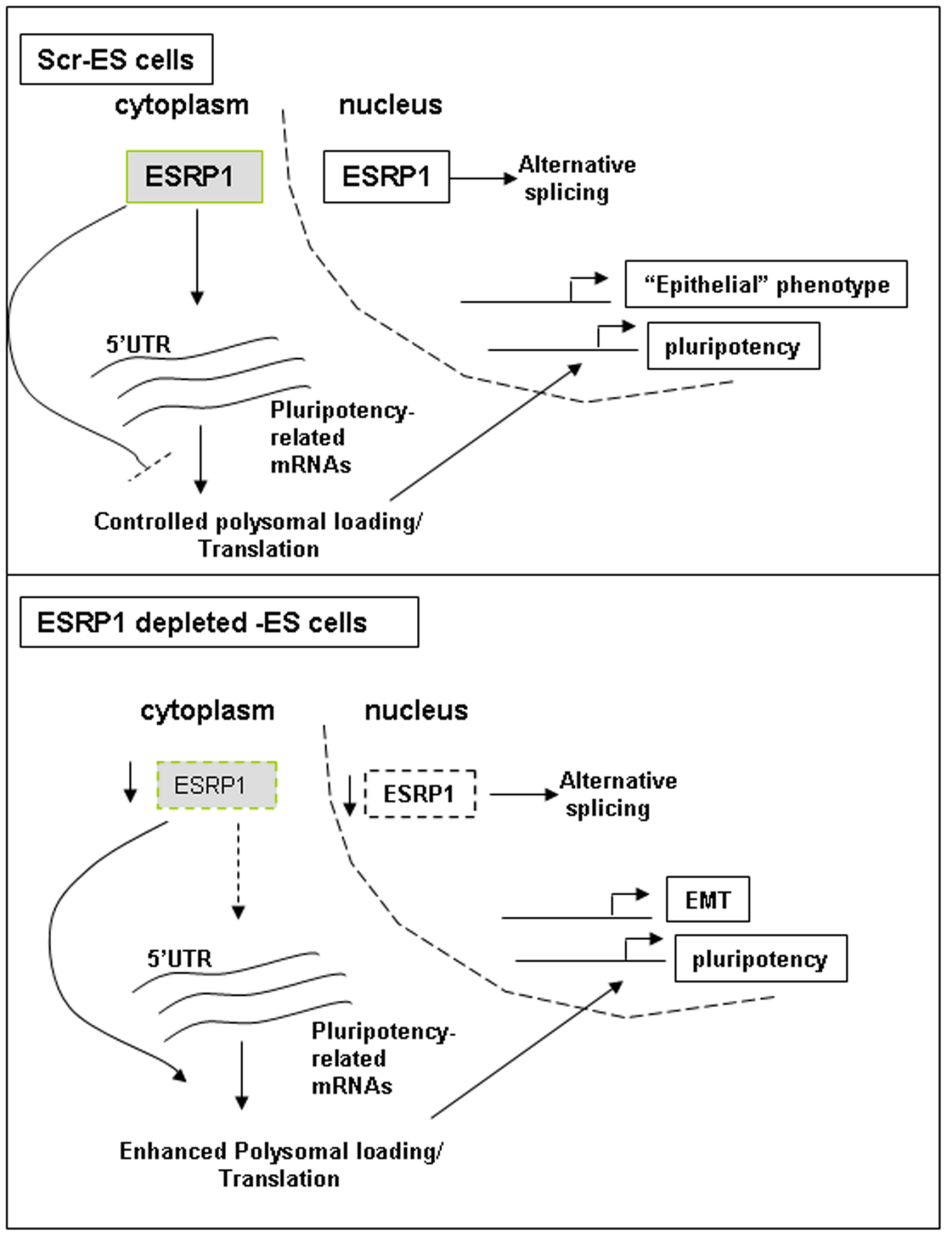
Model for the role of ESRP1 in regulating pluripotency. Under basal conditions (upper panel), ESRP1 in the cytoplasm binds to the 5′UTR of mRNAs of core pluripotency factors and negatively regulates their polysomal loading and translation. In the nucleus, the well-known role of ESRP1 in alternative splicing ensures an epithelial-like phenotype in these cells. The ES cells are thus in a differentiation-poised state. When ESRP1 is depleted (lower panel), the check imposed by this protein on the translation of core pluripotency factors mRNAs is removed, and there is excess translation of the latter resulting in impaired early differentiation of these ES cells. These cells also undergo an EMT-like change.

Another important question is how does ESRP1 work at the molecular level? The increased levels of pluripotency factors that we detected in Esrp1-depleted cells as well as the luciferase reporter assays, together with the previous information on ESRP1 functions, strongly suggest that it may influence the expression of these genes by directly binding to their mRNA. Consistently, we found that the mRNA of c-Myc as well as the mRNA of Oct4, and Sox2 can be specifically co-immunoprecipitated with ESRP1. This interaction does not affect the decay rate of the studied mRNAs (Figure S7), suggesting that ESRP1 probably does not bind to the 3′UTR of these mRNAs and does not affect mRNA stability [48]. The alternative possibility that ESRP1 may primarily regulate translation was already supported by previous evidence. Indeed, it has been shown that ectopic expression of ESRP1 results in moderate changes in polysomal loading of a number of genes and that it can exert a differential effect on reporter RNA translation mediated by various 5′UTRs [32]. The degree of 5′UTR-mediated translational inhibition by ESRP1 is strongly dependent on the complexity of the secondary structure of the 5′UTRs. Esrp1 was also shown to control the translation of the oncogene and pluripotency factor c-Myc [32]. Consistent with this hypothesis, we found that, in Esrp1-depleted cells, the polysomal loading of pluripotency genes is significantly increased and that ESRP1 is not associated with polysomes. These results strongly support a scenario in which ESRP1 reduces the levels of pluripotency factors primarily by binding to their mRNAs and thus preventing their association to active ribosomes. As the pluripotency transcription factors are known to form part of a feed forward auto-regulatory loop, even the moderate rise in their protein levels may result in increased transcription as observed upon ESRP1 depletion in mouse ES cells (Figure 8) [46]. However, we cannot rule out the possibility that ESRP1 may prevent translation initiation, like for e.g., the physical binding of Esrp1 to these mRNAs may interfere with the binding of certain elongation initiation factors that form the initiation complex. We also cannot exclude that ESRP1 is acting, in parallel, on alternative splicing of other factors of the extended transcriptional network and thus influencing the self-renewing capacity of ES cells.

## Conclusion

Our results strongly support the role of ESRP1 as a physiological regulator of pluripotency-related factors in mouse ES cells. The expression of ESRP1 in other stem cells, of both mouse and human origin, pinpoints the importance of this RBP in stem cell biology.

## Supporting Information

Figure S1 **Two ShRNAs versus Esrp1 gave similar results.** A. Methylene blue and ALP staining of ES cells after infection and puromycin selection in the presence of LIF. NT are non-infected controls; ES cell colonies from Scr control and from Esrp1-depletion with two different ShRNAs versus Esrp1 (E2 and E4) are shown. B. Methylene blue staining of 2000 cells plated at passage 4 on gelatin 7 days post-plating with or without LIF. ES cell colonies from Scr control and from Esrp1-depletion with two different ShRNAs versus Esrp1 (E2 and E4) are shown. C. MTT assay performed 5 days after plating of Scr and Esrp1-depleted (E2 and E4) ES cells on gelatin. Bars indicate mean absorbance at 540 nm (n = 6). D. PCR analyis of CD44 isoforms (CD44 variable(v) and CD44 standard(s)) in NT and Scr ES cells versus Esrp1-depleted (E2 and E4) ES cells. Esrp1 and Oct4 expression was also analysed and normalised to Actin. E. qRT-PCR analysis of FGFR2 IIIc/IIIb ratio in ES cells depleted for Esrp1 with another ShRNA (E4) compared to Scr cells. RQ is relative quantity.
(TIF)

Figure S2 **Esrp-1-depleted ES cells are pluripotent.** Phase contrast images of Scr and Esrp1-depleted ES cell colonies grown on inactivated Mefs. Lower panels show immunofluorescence staining for Oct4 and Nanog. Scale bar is 20 µm.
(TIF)

Figure S3 **Analysis of Esrp1-depleted v6.5 ES cells.** Fractionation of nuclear and cytoplasmic proteins of Scr and Esrp1-depleted ES cells were analysed for the abundance of ESRP1. A representative Western blot is shown. Oct4 was mainly nuclear. Blots were normalised with Actin and Lamin A/C.
(TIF)

Figure S4 **Correct expression of mutated ESRP1.** A. Western blot analysis showing expression of mutated ESRP1-GFP compared to wild type ESRP1-GFP and empty vector using anti-GFP antibody. B. qRT-PCR analysis of the FGFR2 IIIc/IIIb ratio upon rescue in Esrp1-depleted v6.5 ES cells. Cells were transfected either with the empty vector (pEm) or with the mutated Esrp1 (Esrp1*). RQ is relative quantity. C. Rescue experiment was performed on ESRP1-depleted (E4) and control Scr E14 ES cells. E4 is another ShRNA wich gave efficient reduction of ESRP1 expression. qRT-PCR analysis shows the reduction in FGFR2 IIIc/IIIb ratio upon introduction of mutated Esrp1 (Esrp1*) in E4 cells. RQ is relative quantity (n = 3).
(TIF)

Figure S5 **Generation of iPS cells from Scr and Esrp1-depleted Mefs.** A. Representative qRT-PCR analysis of Esrp1, Oct4, Nanog and Sox2 expression at different time points during the reprogramming process. B. Representative fluorescence images for CDy1 probe (red) of iPS colonies generated from OSK-infected Mefs only (NT) and those double-infected either with OSK and lentivirus expressing short hairpin versus Scr or Esrp1. Bars show mean counts of colonies per dish. Scale bar is 100 µm. C. Oct4 staining of iPS cells generated from Esrp1-depleted Mefs versus non-infected (NT) or Scr controls. Scale bar is 100 µm.
(TIF)

Figure S6 **Differentiative potential of iPS cells generated from Mefs infected with lentivirus harbouring ShRNA against Scr or Esrp1.** A. qRTPCR analysis of EBs generated for the indicated time points shows that all three iPS cell types (NT, Scr and E2) differentiate into the 3 germ layers. This graph is representative of 2 independent analyses. B. 5×10^5^ iPS cells were injected subcutaneously in five NOD-scid mice. Tumors were sought after 4 weeks. Hematoxylin/eosin staining of the teratoma sections reveal the presence of the 3 germ layers.
(TIF)

Figure S7 **Histological analysis of teratomas.** Hematoxylin/eosin (H/E) staining of sections of teratomas generated from ESRP1-depleted ES cells compared to those derived from Scr ES cells. Asterisks show representative neuroepithelium shown in inset. PCNA staining shows that ESRP1-depleted teratomas have larger proliferating neuroepithelial areas compared to Scr teratomas. Arrows show neuroepithelium.
(TIF)

Figure S8 **Analysis of ESRP1 expression in human stem/progenitor cells.** CD133+ kidney progenitor cells (KPC) [26] express ESRP1 while kidney cancer stem cells (KCSC) [27] do not.
(TIF)

Figure S9 **RNA-immunoprecipitation in Scr ES cells.** qRT-PCR analysis of mRNA eluted from RIP in Scr ES cells shows that there was little binding to preimmune IgG for Oct4, Sox2 and cMyc mRNAs versus anti-ESRP1 antibody. This graph is representative of 2 independent experiments.
(TIF)

Figure S10 **mRNA decay rates of pluripotency-related mRNAs upon Esrp1 depletion.** qRT-PCR analysis of the percentage of Oct4, Nanog, Sox2, c-Myc and Esrp1 mRNA remaining in the ES cells after actinomycin D treatment for the indicated time points (n = 6).
(TIF)

Table S1 **Primers used for PCR and qRT-PCR, and UPL probes used in this study.**
(DOC)

Table S2 **Primers used for mutagenesis of Esrp1 cDNA at ShRNA binding site.**
(DOC)

Table S3 **Antibodies used in this study.**
(DOC)

Table S4 **Stem cell-specific co-expression analysis reveals genes that are co-expressed with Oct4, Sall4, L1TD1 and Dppa4.**
(DOC)

Table S5 **qRT-PCR analysis following RNA-IP with anti-ESRP1 antibody.** Results show relative quantity of mRNA of each gene immunoprecipitating with anti-ESRP1 antibody in primary Mefs, or using ES cells infected with lentivirus harbouring short hairpin against Scr or GFP versus short hairpin against Esrp1.
(DOC)

File S1 **Supplementary Materials and Methods.**
(DOC)
