# Supplementary figures and images for: The RNA Binding Protein ESRP1 Fine-Tunes the Expression of Pluripotency-Related Factors in Mouse Embryonic Stem Cells
Source: PLoS One. 2013 Aug 27;8(8):e72300. doi: 10.1371/journal.pone.0072300 (PMC3755004; doi:10.1371/journal.pone.0072300)

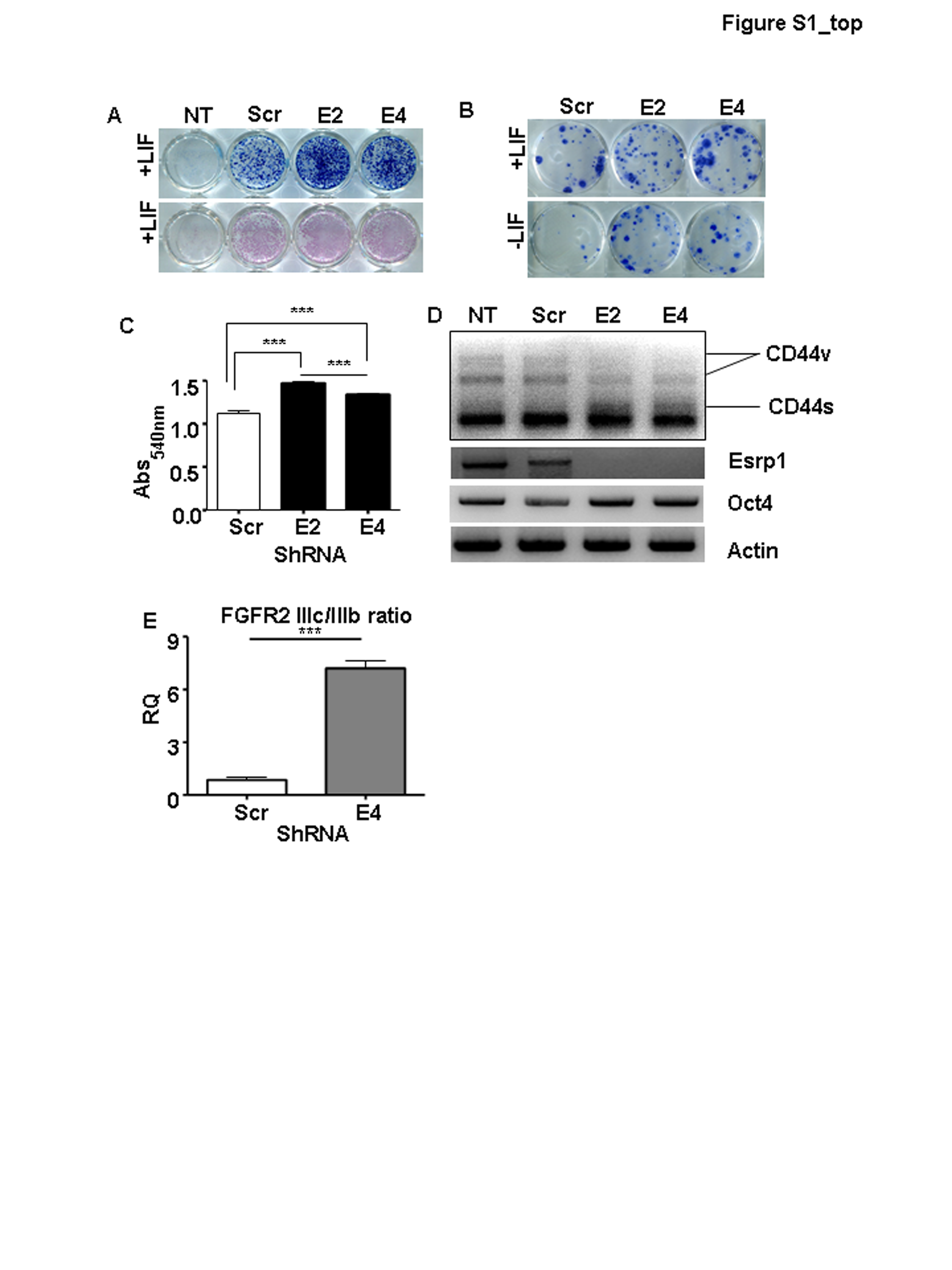

Supplement: Figure S1 — Two ShRNAs versus Esrp1 gave similar results. A. Methylene blue and ALP staining of ES cells after infection and puromycin selection in the presence of LIF. NT are non-infected controls; ES cell colonies from Scr control and from Esrp1-depletion with two different ShRNAs versus Esrp1 (E2 and E4) are shown. B. Methylene blue staining of 2000 cells plated at passage 4 on gelatin 7 days post-plating with or without LIF. ES cell colonies from Scr control and from Esrp1-depletion with two different ShRNAs versus Esrp1 (E2 and E4) are shown. C. MTT assay performed 5 days after plating of Scr and Esrp1-depleted (E2 and E4) ES cells on gelatin. Bars indicate mean absorbance at 540 nm (n = 6). D. PCR analyis of CD44 isoforms (CD44 variable(v) and CD44 standard(s)) in NT and Scr ES cells versus Esrp1-depleted (E2 and E4) ES cells. Esrp1 and Oct4 expression was also analysed and normalised to Actin. E. qRT-PCR analysis of FGFR2 IIIc/IIIb ratio in ES cells depleted for Esrp1 with another ShRNA (E4) compared to Scr cells. RQ is relative quantity. (TIF) [file pone.0072300.s001.tif]

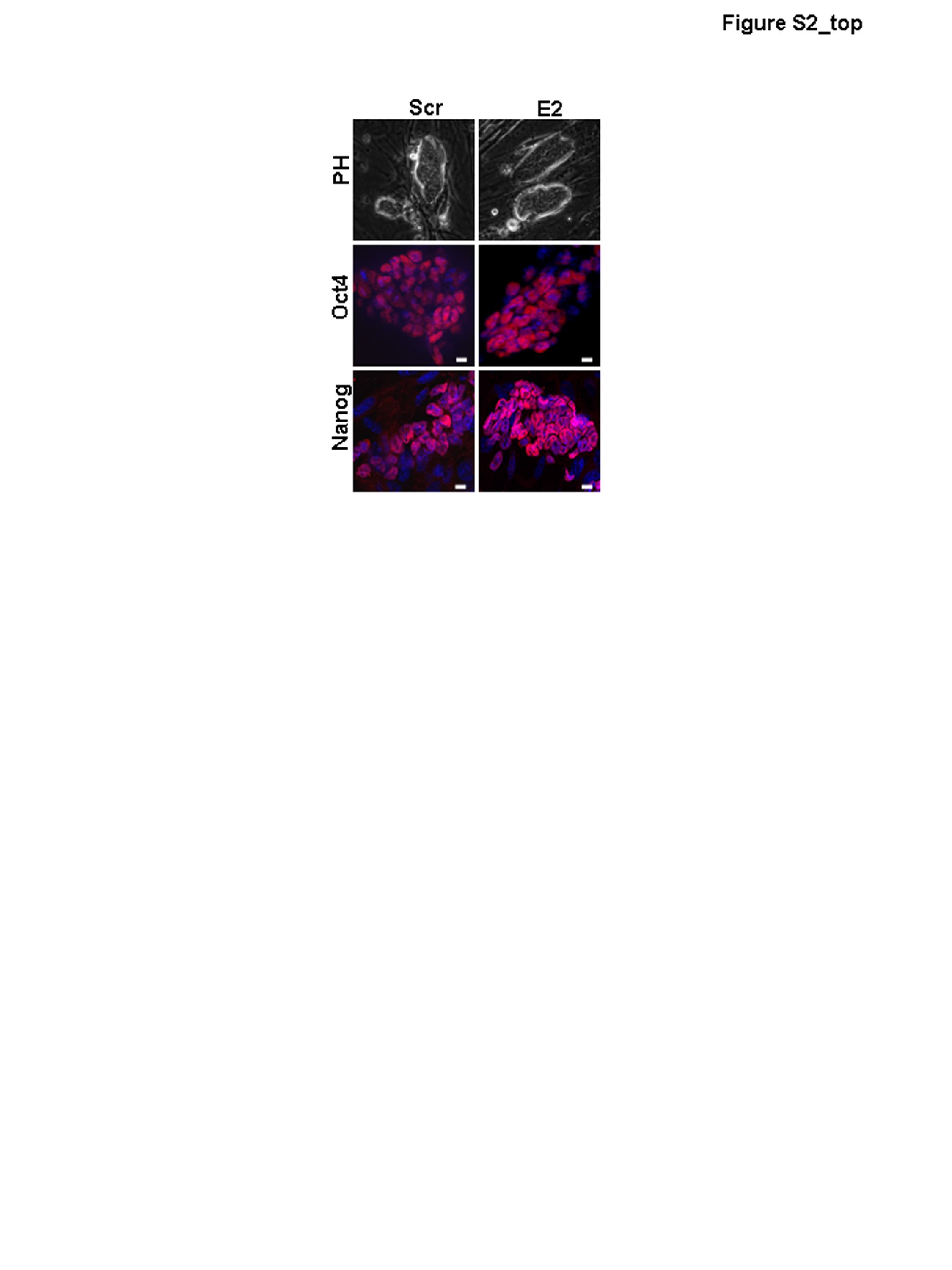

Supplement: Figure S2 — Esrp-1-depleted ES cells are pluripotent. Phase contrast images of Scr and Esrp1-depleted ES cell colonies grown on inactivated Mefs. Lower panels show immunofluorescence staining for Oct4 and Nanog. Scale bar is 20 µm. (TIF) [file pone.0072300.s002.tif]

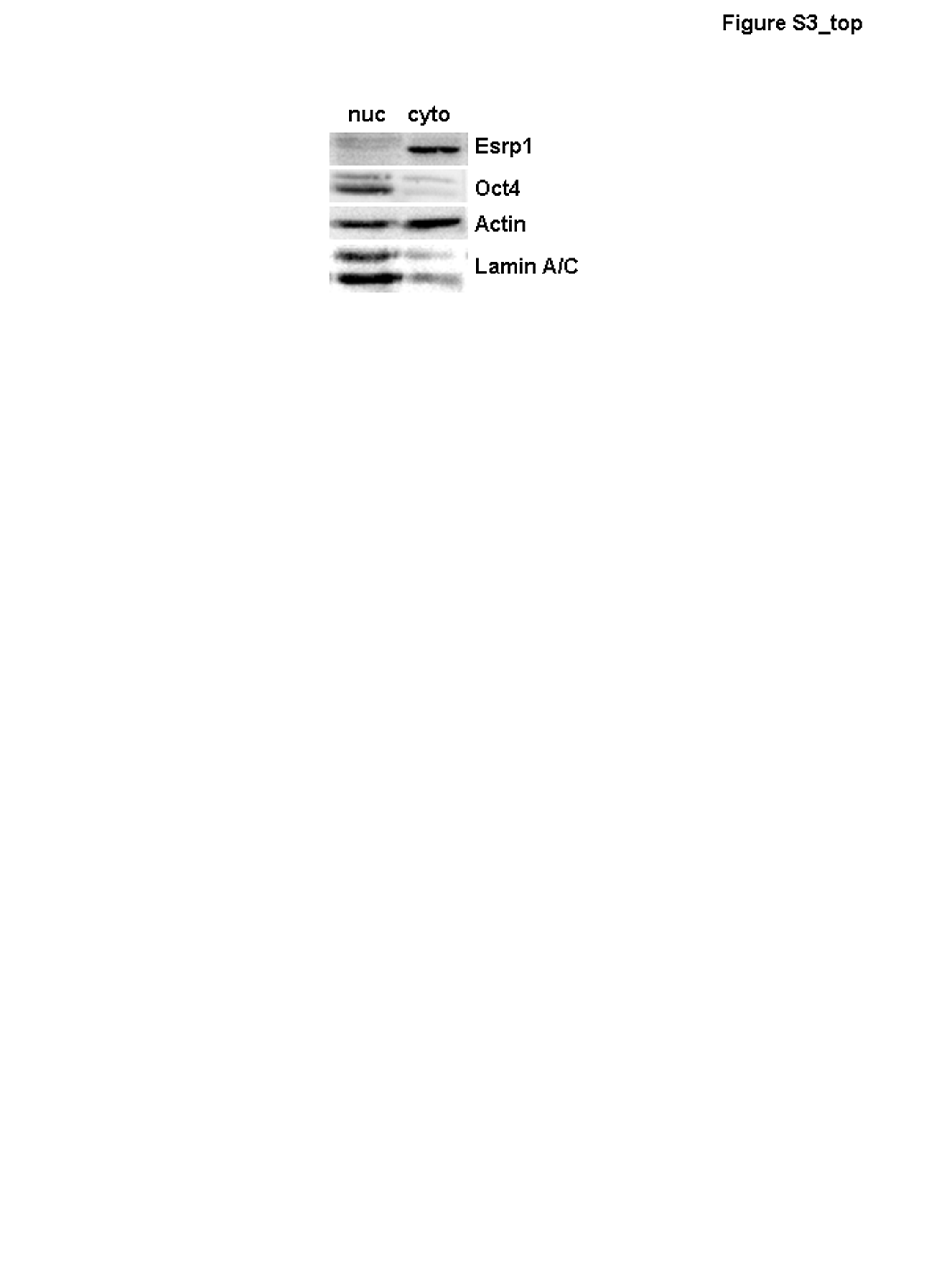

Supplement: Figure S3 — Analysis of Esrp1-depleted v6.5 ES cells. Fractionation of nuclear and cytoplasmic proteins of Scr and Esrp1-depleted ES cells were analysed for the abundance of ESRP1. A representative Western blot is shown. Oct4 was mainly nuclear. Blots were normalised with Actin and Lamin A/C. (TIF) [file pone.0072300.s003.tif]

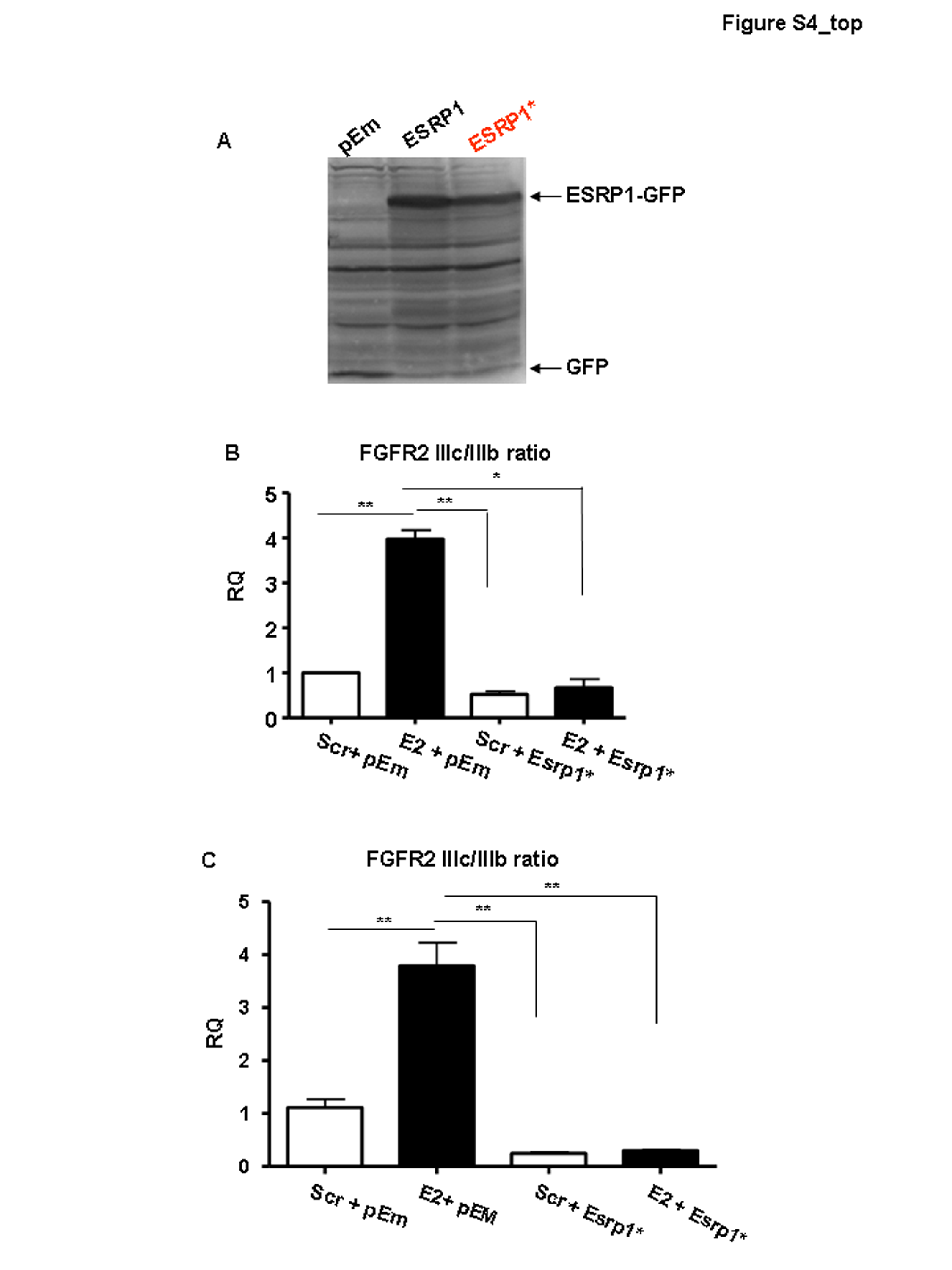

Supplement: Figure S4 — Correct expression of mutated ESRP1. A. Western blot analysis showing expression of mutated ESRP1-GFP compared to wild type ESRP1-GFP and empty vector using anti-GFP antibody. B. qRT-PCR analysis of the FGFR2 IIIc/IIIb ratio upon rescue in Esrp1-depleted v6.5 ES cells. Cells were transfected either with the empty vector (pEm) or with the mutated Esrp1 (Esrp1*). RQ is relative quantity. C. Rescue experiment was performed on ESRP1-depleted (E4) and control Scr E14 ES cells. E4 is another ShRNA wich gave efficient reduction of ESRP1 expression. qRT-PCR analysis shows the reduction in FGFR2 IIIc/IIIb ratio upon introduction of mutated Esrp1 (Esrp1*) in E4 cells. RQ is relative quantity (n = 3). (TIF) [file pone.0072300.s004.tif]

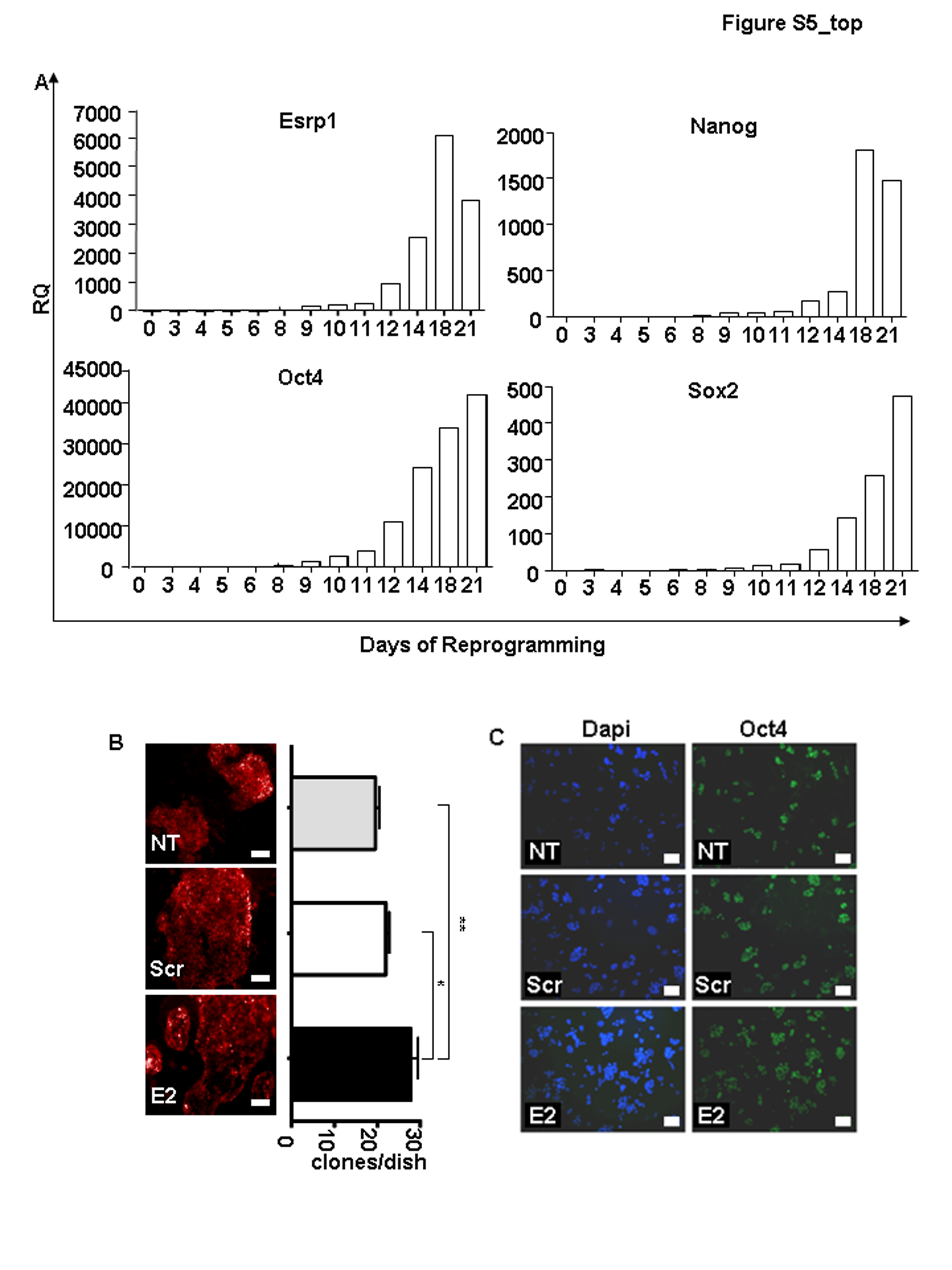

Supplement: Figure S5 — Generation of iPS cells from Scr and Esrp1-depleted Mefs. A. Representative qRT-PCR analysis of Esrp1, Oct4, Nanog and Sox2 expression at different time points during the reprogramming process. B. Representative fluorescence images for CDy1 probe (red) of iPS colonies generated from OSK-infected Mefs only (NT) and those double-infected either with OSK and lentivirus expressing short hairpin versus Scr or Esrp1. Bars show mean counts of colonies per dish. Scale bar is 100 µm. C. Oct4 staining of iPS cells generated from Esrp1-depleted Mefs versus non-infected (NT) or Scr controls. Scale bar is 100 µm. (TIF) [file pone.0072300.s005.tif]

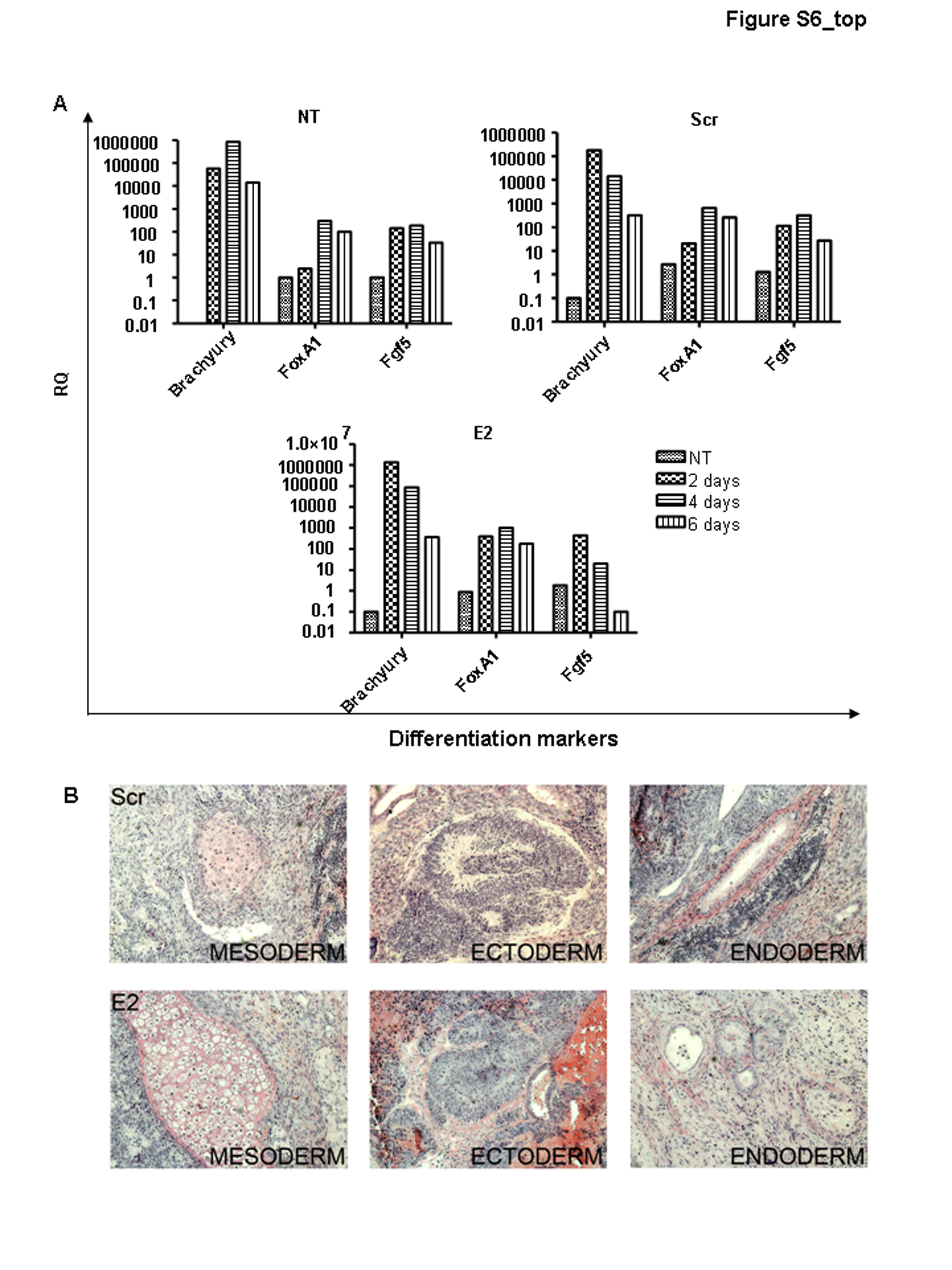

Supplement: Figure S6 — Differentiative potential of iPS cells generated from Mefs infected with lentivirus harbouring ShRNA against Scr or Esrp1. A. qRTPCR analysis of EBs generated for the indicated time points shows that all three iPS cell types (NT, Scr and E2) differentiate into the 3 germ layers. This graph is representative of 2 independent analyses. B. 5×10^5^ iPS cells were injected subcutaneously in five NOD-scid mice. Tumors were sought after 4 weeks. Hematoxylin/eosin staining of the teratoma sections reveal the presence of the 3 germ layers. (TIF) [file pone.0072300.s006.tif]

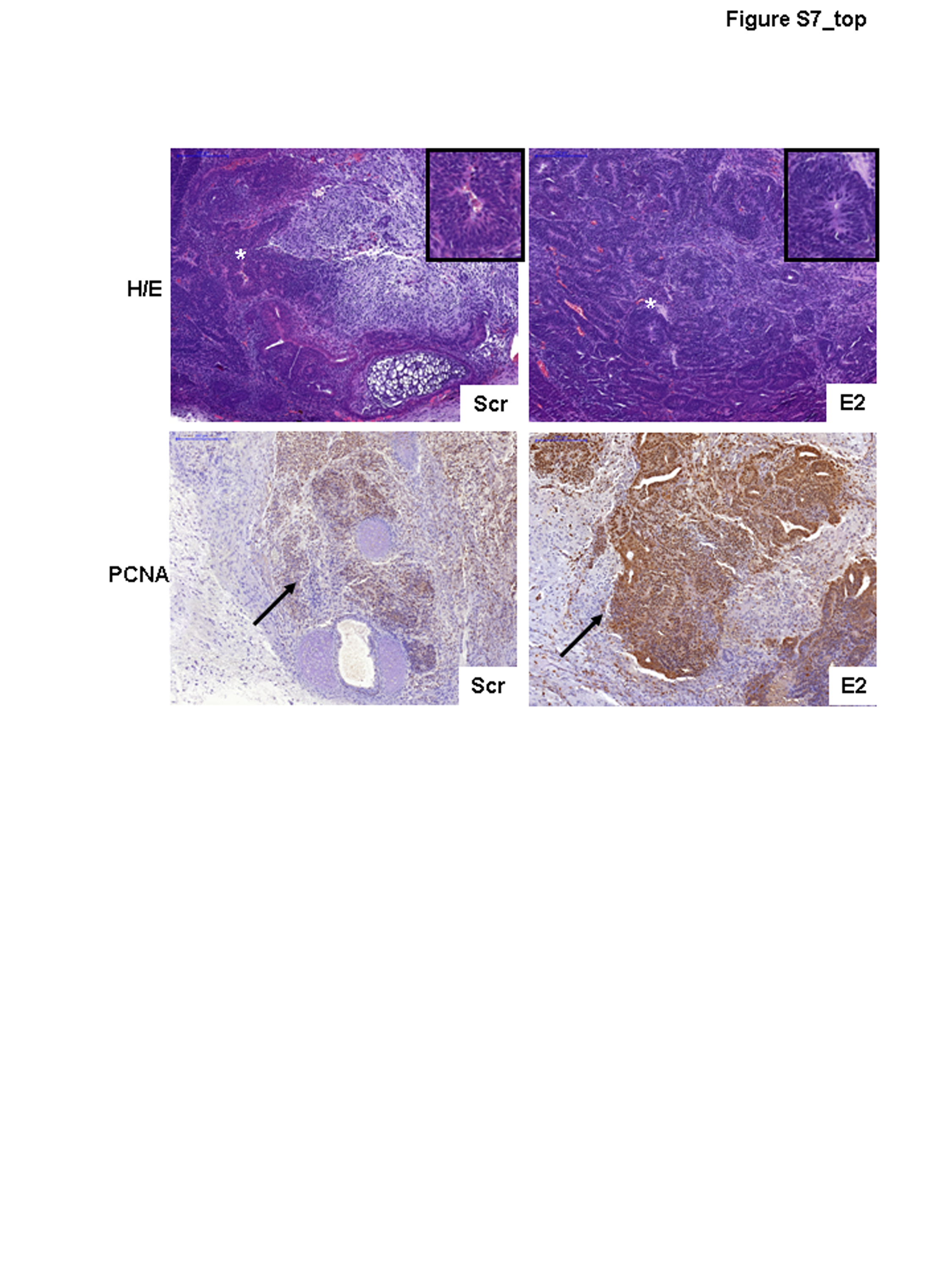

Supplement: Figure S7 — Histological analysis of teratomas. Hematoxylin/eosin (H/E) staining of sections of teratomas generated from ESRP1-depleted ES cells compared to those derived from Scr ES cells. Asterisks show representative neuroepithelium shown in inset. PCNA staining shows that ESRP1-depleted teratomas have larger proliferating neuroepithelial areas compared to Scr teratomas. Arrows show neuroepithelium. (TIF) [file pone.0072300.s007.tif]

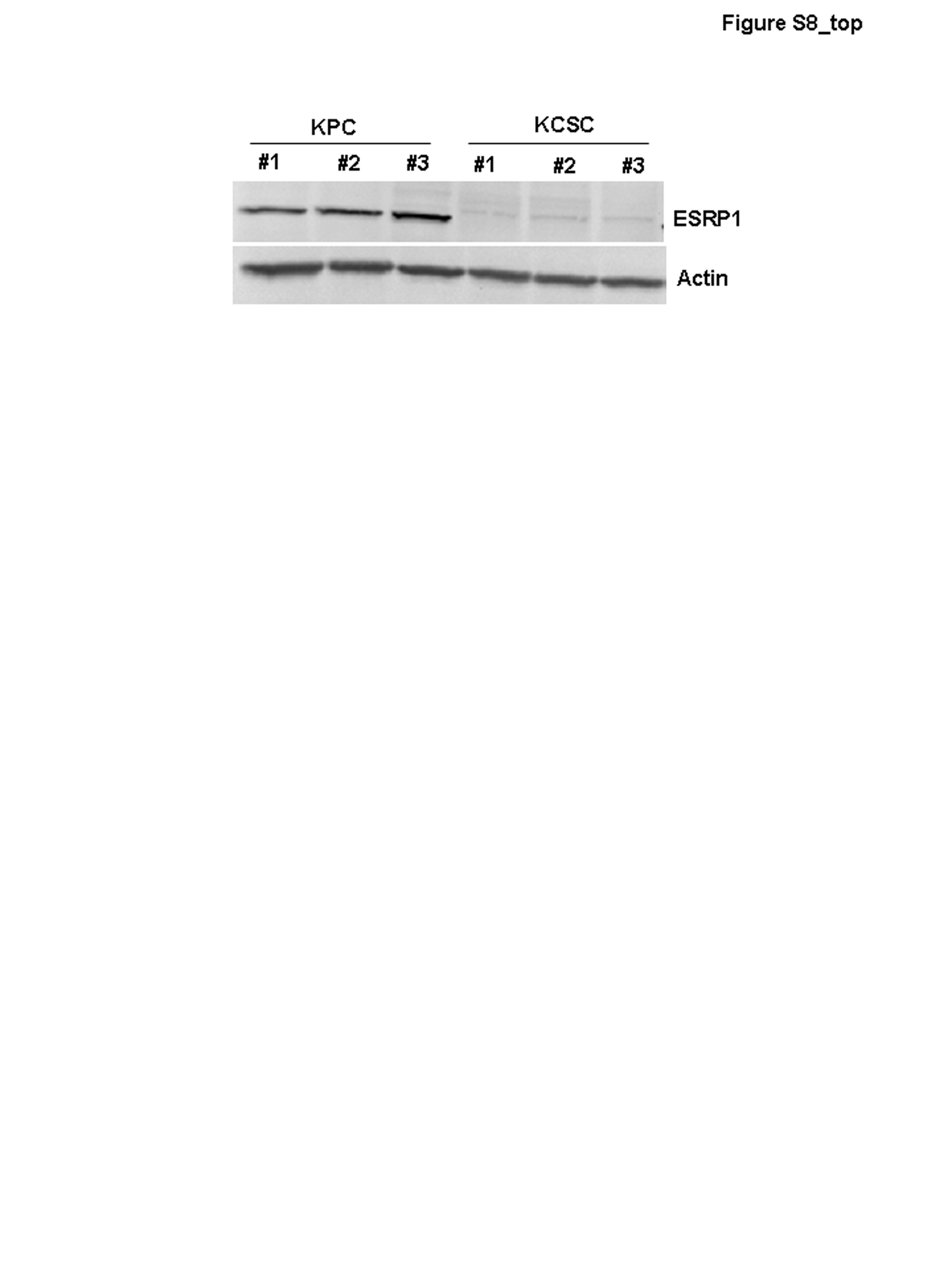

Supplement: Figure S8 — Analysis of ESRP1 expression in human stem/progenitor cells. CD133+ kidney progenitor cells (KPC) [26] express ESRP1 while kidney cancer stem cells (KCSC) [27] do not. (TIF) [file pone.0072300.s008.tif]

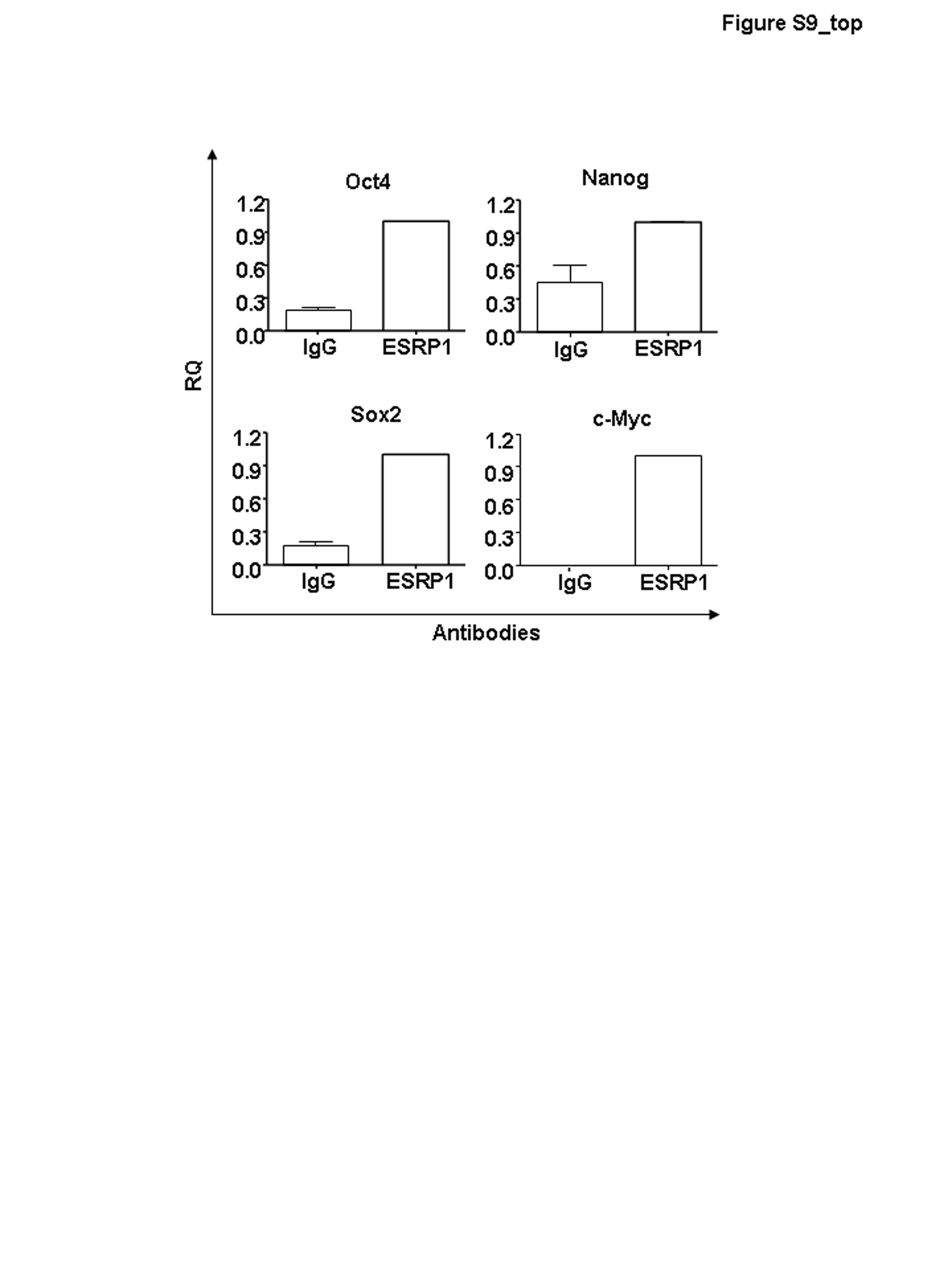

Supplement: Figure S9 — RNA-immunoprecipitation in Scr ES cells. qRT-PCR analysis of mRNA eluted from RIP in Scr ES cells shows that there was little binding to preimmune IgG for Oct4, Sox2 and cMyc mRNAs versus anti-ESRP1 antibody. This graph is representative of 2 independent experiments. (TIF) [file pone.0072300.s009.tif]

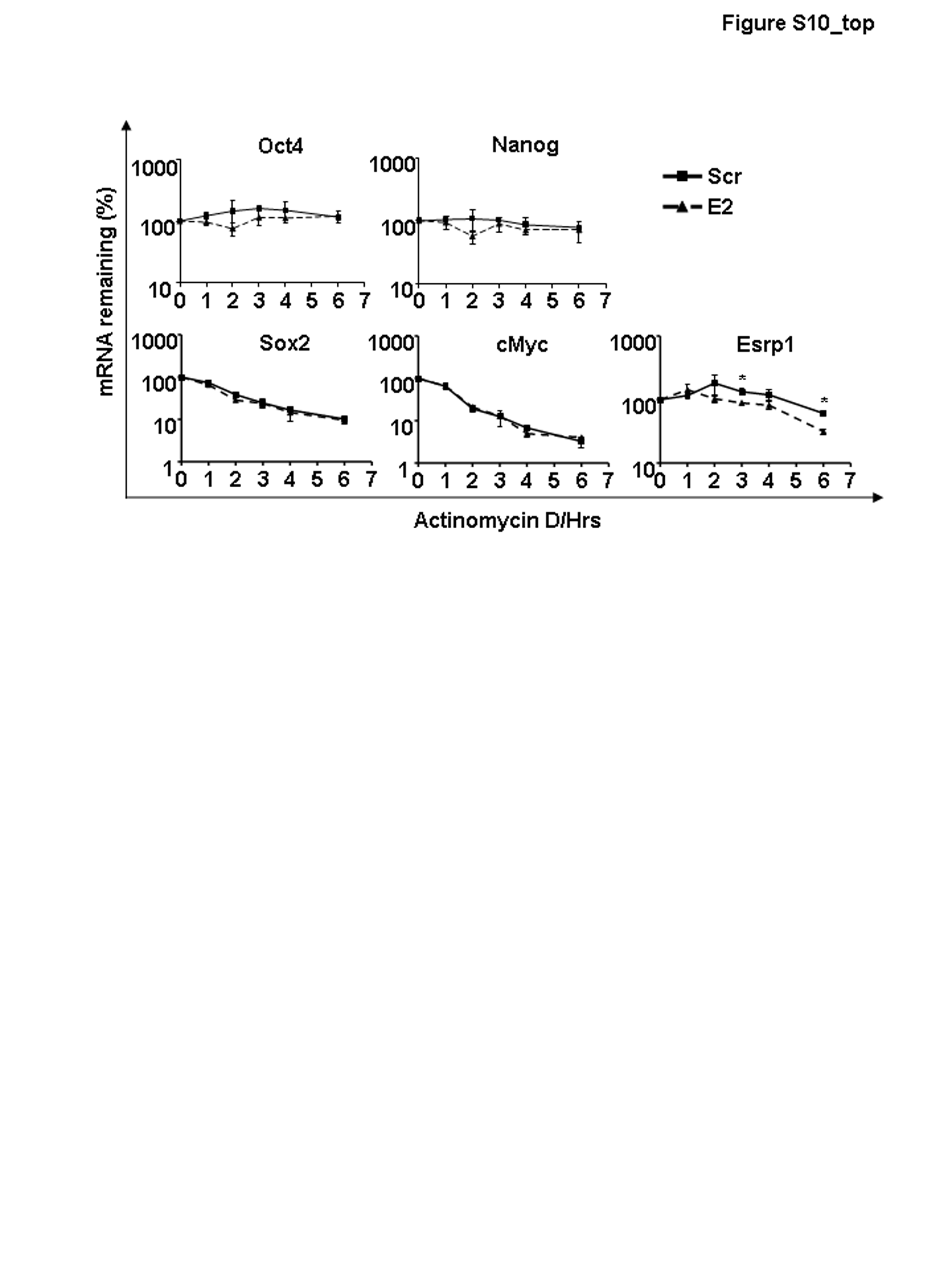

Supplement: Figure S10 — mRNA decay rates of pluripotency-related mRNAs upon Esrp1 depletion. qRT-PCR analysis of the percentage of Oct4, Nanog, Sox2, c-Myc and Esrp1 mRNA remaining in the ES cells after actinomycin D treatment for the indicated time points (n = 6). (TIF) [file pone.0072300.s010.tif]
