# Supplementary material for: The RNA Binding Protein ESRP1 Fine-Tunes the Expression of Pluripotency-Related Factors in Mouse Embryonic Stem Cells
Source: PLoS One. 2013 Aug 27;8(8):e72300. doi: 10.1371/journal.pone.0072300 (PMC3755004; doi:10.1371/journal.pone.0072300)
Supplement: Table S1 — Primers used for PCR and qRT-PCR, and UPL probes used in this study. (DOC) [file pone.0072300.s011.doc]

Table S1

qR-PCR primers and probes

| Gene Name | Primers 5’-3’ | UPL Probe (Roche) |
| --- | --- | --- |
| Esrp1 | Left: cccaccgccatgtaagtt  Right: gtgggagctgggaatgtgta | #52 |
| Oct4 | Left: gttggagaaggtggaaccaa  Right: ctccttctgcagggctttc | #95 |
| Nanog | Left: ttcttgcttacaagggtctgc  Right: agaggaagggcgaggaga | #110 |
| c-Myc | Left: cctagtgctgcatgaggaga  Right: tccacagacaccacatcaattt | #77 |
| Sox2 | Left: tgctgcctctttaagactaggg  Right: tcgggctccaaacttctct | #91 |
| Brachyury | Left: cagcccacctactggctcta  Right: gagcctggggtgatggta | #100 |
| Cdx2 | Left: caccatcaggaggaaaagtga  Right: ctgcggttctgaaaccaaat | #34 |
| Fgf5 | Left: aaaacctggtgcaccctaga  Right: catcacattcccgaattaagc | #29 |
| Klf4 | Left: ccagatgcagtcacaagtcc  Right: gaccttcttcccctctttgg | #82 |
| Lin28 | Left: gagtccaggatgattccaaga  Right: tgctctgacagtaatggcactt | # 73 |
| Snai1 | Left: gtctgcacgacctgtggaa  Right: caggagaatggcttctcacc | #71 |
| Slug | Left: tgcaagatctgtggcaagg  Right: cagtgagggcaagagaaagg | #71 |
| Vimentin | Left: ccaaccttttcttccctgaac  Right: ttgagtgggtgtcaaccaga | #109 |
| E-cadherin | Left: atcctcgccctgctgatt  Right: accaccgttctcctccgta | #18 |
| FGFR2 IIIb | Left: tcctgcccaaacagcaag  Right: gaagacccctatgcagtaaatagc | #60 |
| FGFR2 IIIc | Left: tgcatggttgacagttctgc  Right: tgcaggcgattaagaagacc | #60 |

PCR primers:

CD44 Fw: 5’ tccttctttatccggagcac

CD44 Rev (exon 11): 5’ agctgctgcttctgctgtact
