# Supplementary material for: The RNA Binding Protein ESRP1 Fine-Tunes the Expression of Pluripotency-Related Factors in Mouse Embryonic Stem Cells
Source: PLoS One. 2013 Aug 27;8(8):e72300. doi: 10.1371/journal.pone.0072300 (PMC3755004; doi:10.1371/journal.pone.0072300)
Supplement: Table S2 — Primers used for mutagenesis of Esrp1 cDNA at ShRNA binding site. (DOC) [file pone.0072300.s012.doc]

Table S2

| **Primers Name** | **5’ to 3’ sequence** |
| --- | --- |
| ESRPmutFW | CATGGGGACCCGGTATATCGAAGTCTACAAAGCAACAGGCG |
| ESRPmutREV | CGCCTGTTGCTTTGTAGACTTCGATATACCGGGTCCCCATG |
