# Supplementary material for: The RNA Binding Protein ESRP1 Fine-Tunes the Expression of Pluripotency-Related Factors in Mouse Embryonic Stem Cells
Source: PLoS One. 2013 Aug 27;8(8):e72300. doi: 10.1371/journal.pone.0072300 (PMC3755004; doi:10.1371/journal.pone.0072300)
Supplement: Table S3 — Antibodies used in this study. (DOC) [file pone.0072300.s013.doc]

Table S3

| Antibody | Catalog number | Manufacturer | Western Blot  (Dilution) | Immunofluorescence  (Dilution) |
| --- | --- | --- | --- | --- |
| Oct4 | ab19857 | Abcam | 1:1000 | 1:100 |
| Sox2 | ab59776 | Abcam | 1:1000 |  |
| Nanog | ab70482 | Abcam | 1:1000 | 1:100 |
| Esrp1 | ab109380 | Abcam | 1:1000 | 1:100 |
| c-Myc | clone 9E10 | In-house | 1:500 |  |
| Lamin A/C | sc-20681 | Santa Cruz Biotechnology | 1:1000 |  |
| GFP | Clone 2G8/C11 | In-house | 1:1000 |  |
| S6 Ribosomal Protein(54D2) | 2317 | Cell signaling | 1:1000 |  |
| Ribosomal Protein L4 (RQ-7) | sc-100838 | Santa Cruz | 1:1000 |  |
| DDX4 / MVH - Primordial Germ Cell Marker | ab13840 | Abcam | 1:1000 |  |
| PCNA | Sc-56 | Santa Cruz |  | 1: 50 |
| Actin | Sc-1616 | Santa Cruz | 1:1000 |  |
