# Supplementary material for: The RNA Binding Protein ESRP1 Fine-Tunes the Expression of Pluripotency-Related Factors in Mouse Embryonic Stem Cells
Source: PLoS One. 2013 Aug 27;8(8):e72300. doi: 10.1371/journal.pone.0072300 (PMC3755004; doi:10.1371/journal.pone.0072300)
Supplement: Table S4 — Stem cell-specific co-expression analysis reveals genes that are co-expressed with Oct4, Sall4, L1TD1 and Dppa4. (DOC) [file pone.0072300.s014.doc]

Table S4
