# Supplementary material for: The RNA Binding Protein ESRP1 Fine-Tunes the Expression of Pluripotency-Related Factors in Mouse Embryonic Stem Cells
Source: PLoS One. 2013 Aug 27;8(8):e72300. doi: 10.1371/journal.pone.0072300 (PMC3755004; doi:10.1371/journal.pone.0072300)
Supplement: Table S5 — qRT-PCR analysis following RNA-IP with anti-ESRP1 antibody. Results show relative quantity of mRNA of each gene immunoprecipitating with anti-ESRP1 antibody in primary Mefs, or using ES cells infected with lentivirus harbouring short hairpin against Scr or GFP versus short hairpin against Esrp1. (DOC) [file pone.0072300.s015.doc]

|  | **Mefs** | **ES cells** | | |
| --- | --- | --- | --- | --- |
| **mRNA** | **NT** | **ShScr** | **ShGFP** | **ShESRP1 (E2)** |
| Oct4* | 0 | 1 | 0,812 | 0,649 |
| Nanog | 0 | 1 | 1,252 | 0,404 |
| Sox2 | 0 | 1 | 0,979 | 0,404 |
| c-Myc | 0,154 | 1 | 0,268 | 0,146 |
| N-myc* | 0,049 | 1 | 1,474 | 0,805 |
| Lin28* | 0,001 | 1 | 1,024 | 0,811 |

Table S5

| Oct4 |  |  |
| --- | --- | --- |
|  |  |  |
