# Supplementary material for: The RNA Binding Protein ESRP1 Fine-Tunes the Expression of Pluripotency-Related Factors in Mouse Embryonic Stem Cells
Source: PLoS One. 2013 Aug 27;8(8):e72300. doi: 10.1371/journal.pone.0072300 (PMC3755004; doi:10.1371/journal.pone.0072300)
Supplement: File S1 — Supplementary Materials and Methods. (DOC) [file pone.0072300.s016.doc]

**SUPPLEMENTARY MATERIALS AND METHODS**

**Cell Culture.**

E14 and v6.5 ES cells as well as GPSCs and iPS cells were grown on mouse embryonic fibroblasts (Mefs) in ES cell medium composed of Dulbecco’s Modified Eagle Medium (DMEM) with Glutamax (Invitrogen), supplemented with 15% heat-inactivated fetal calf serum (FCS, Hyclone, Thermo Scientific), 150µM of monothioglycerol (Sigma), 100µg/ml penicillin/streptomycin (PS, Invitrogen), Non essential amino acids (NEAA, Invitrogen), Sodium pyruvate (Invitrogen) and 1,000 U/ml of leukaemia inhibitory factor (LIF, in-house). ES cell were split every two days with daily medium change. In all experiments, cells were used within three to five passages after initial thawing. Cultures were maintained at 37°C with 5% CO_2_. EBs medium was composed of Iscove's Modified Dulbecco's Medium (IMDM) with Glutamax (Invitrogen), supplemented with 9% FCS and NEAA, sodium pyruvate, PS, monothioglycerol (Sigma) as described above.

For all the experiments, except teratomas and immunofluorescence analysis, ES cells were trypsinized, depleted of feeders by 30 minutes preplating.

**SSC cultures**

For initiation of SSC cultures, αv-integrin (CD51)^neg^, CD45^neg^, and Thy1.2(CD90.2)^high^ testis cells from juvenile mice (10 to 14 days postnatal) were flow sorted (MoFLo XDP, Beckman Coulter) and plated onto Mefs as previously described [1]. Antibodies for flow sorting were obtained from Biolegend. Medium for SSC culture was prepared as previously described [2] with some modifications: the medium was supplemented with 10 ng/ml IGF-I (Peprotech) and insulin, transferrin and selenium were substituted with ITS from Cellgro. For the preparation of adult SSC, testicular cells were incubated with a biotin anti-EPCAM antibody (BD Pharmingen™), followed incubation with Streptavidin magnetic beads and MACS cell sorting (Miltenyi Biotec) according to the manufacturer’s instructions. Media was changed daily and SSCs passaged on a weekly basis.

**Lentivirus production**

Five ShRNA from Openbiosystems (TRCN0000127334- TRCN0000127338) were analyzed for efficient knockdown of Esrp1. Briefly, each vector was transiently delivered into mouse ES cells for 3 days and total RNA extracted as described below and analyzed by qRT-PCR. Two ShRNA sequences (named herein, E2 and E4) gave the best results and were used for lentiviral production as described [3].

**Lentiviral infection of mES cells**

E14 ES cells were preplated on gelatinized plates for 30 minutes to remove Mefs. 2x10^5^ ES cells were then infected in suspension with 1:1 ratio of lentiviral supernatant: ES cell media in the presence of 8μg/mL polybrene (Sigma, H9268-10G). After 3 hours, cells were seeded onto gelatinized 6 well plates. Media was changed the day after and puromycin selection started 48-72 hours after infection.

**Transient transfection**

ES cells (Scr and E2) were trypsinised and preplated for 30 minutes to remove Mef feeder cells. Fifty thousand ES cells were plated per well in a gelatin-coated 24-well dish. Reverse transfection was performed using Lipofectamine 2000 (Invitrogen) following the manufacturer’s instructions. For the rescue experiment, pIBX-C-FF-EmGFP (pEm empty vector) and pIBX-C-FF-EmGFP-B-ESRP1-2A (mutagenised Esrp1 cDNA containing vector, Esrp1*) were transiently transfected into ES cells and lysed after 3 days.

**Colony forming assay**

Cells were plated onto 0.2% gelatin for 5 days, cultured in the presence or absence of LIF and stained for alkaline phosphatase using Alkaline Phosphatase detection kit (Millipore) or with methylene blue for colony scoring.

**Proliferation assays**

Two thousand ES cells (Scr and Esrp1-depleted) per well were cultured in gelatinized 96-well plates for 1, 2 and 5 days. ES cell medium was daily changed. MTT assay was performed according to the manufacturer’s instructions (Roche Applied Science) and measured on a GloMax®-Multi
Microplate reader (Promega).

**qRT-PCR analysis**

Total RNA extracted using the mini-to-midi RNA extraction kit (Invitrogen). Target gene expression was analyzed by qRT-PCR (Table S3) and normalized to endogenous 18s or actin expression as previously described[4].

**Immunofluorescence**

Cells were fixed in 2% paraformaldehyde for 10 min at RT, washed with PBS, and permeabilized for 10 min with 0.5% Triton X-100 and blocked for 1 hour in blocking buffer (1% BSA, 10% goat serum, 0.1% Triton X-100 in PBS). Primary antibodies (Table S4) were diluted in blocking buffer, and incubated overnight at 4°C. Alexa Fluor-conjugated secondary antibodies were incubated 1h at RT. DNA was visualized using DAPI (0.5µg/ml). Images were acquired using a Zeiss microscope (Apotome software) or Leica TCS SP5 microscope for confocal imaging.

**Western Blotting**

To analyse the expression of OCT4, NANOG and SOX2, nuclear extracts were obtained using Isotonic Buffer (20 mM HEPES,100 mM NaCl, 250 mM Sucrose, 5mM MgCl2, a cocktail of protease inhibitors (Roche) and DTT) followed by lysis in 1ml of ice-cold RIP buffer (100 mM KCl, 5 mM MgCl2, 10 mM HEPES, 0.5 % NP-40, 1 mM DTT, 0.5 mM CaCl2, a cocktail of protease inhibitors (Roche). Twenty-five micrograms of protein was separated on a 12% SDS-PAGE, transferred to nitrocellulose membrane and incubated overnight with the respective antibodies at 4°C. Anti-rabbit secondary antibody was added for one hour at RT and developed with an in-house ECL system.

Total protein was extracted using TENT buffer and inhibitor of proteases was employed for the analysis of ESRP1.

For analysis of human ESRP1 expression in CD133+ kidney progenitor cells (KPC)[5] and kidney cancer stem cells (KCSC)[6], protein extracts from cells (methods described in the cited references) were generously provided by B. Bussolati.

**RNA-immunoprecipitation**

Cytoplasmic extract was obtained by incubating the ES cells for 5 min in cold isotonic buffer (20 mM HEPES,100 mM NaCl, 250 mM Sucrose, 5mM MgCl2, a cocktail of protease inhibitors (Roche) and RNAse inhibitor (Promega) and DTT. The lysates were precleared for 1 hour at 4°C using sepharose protein A beads. Anti-ESRP1 antibody or rabbit IgG was added to the precleared lysates overnight at 4°C and the day after, sepharose A beads were added for 3 hours at 4°C. After washing, the beads were resuspended in Trizol and RNA extracted according to the manufacturer’s protocol (Invitrogen).

**RNA stability assays**

Cells were cultured for three days in 60mm gelatin-coated dishes as described above. Treatments with actinomycin D were performed in reverse: at 6h, 4h, 3h, 2h and 1h time points, the medium was replaced by ES medium supplemented with 2.5µg/ml of actinomycin D (Sigma). At t=0 time point, cells were lysed and total RNA extracted using the mini-to-midi RNA extraction kit (Invitrogen). Target gene expression was analyzed by qRT-PCR and normalized to endogenous actin expression.

**Polysome preparation**

Polysomes were prepared by means of density gradient centrifugation as follows. Cells were grown to subconfluency and cycloheximide (CHX) was added to media to a final concentration of 100ug/ml for 10 min. Cells were then trypsinized and lyzed with Lysis Buffer (20 mM Tris-Hcl, 5 mM MgCl2, 150 mM NaCl, 1% TritonX-100, 1% Deoxycholate, 2.5 mM DTT, 200 U/ml RNase inhibitor, 100ug/ml cycloheximide) for 5min. Nuclei and mitochondria were pelleted and supernatants were poured on top of a 10-50% sucrose gradient. After spinning at 36 000 rpm for 3 hours at 4^o^C (Beckman Ultracentrifuge), tubes were run on an ISCO fractionating machine as suggested by its protocol and fractions collected for further analysis.

### Induced pluripotent stem (iPS) cells generation

### Primary Mefs were infected with lentivirus harbouring Sh versus Scr or E2. After puromycin selection, cells were counted and iPS cells were generated with OKS vectors essentially as previously described[7]. RNA was extracted at the indicated time points for qRT-PCR analysis. Colonies formed were stained with CDy1 dye or OCT4 for pluripotency determination[8,9]. Teratomas and EBs were generated as described in the main text. Briefly, 5x10^5^ iPS cells were injected subcutaneously in five NOD-scid mice. Tumors were sought after 4 weeks.

**Supplementary references**

1. Hobbs RM, Seandel M, Falciatori I, Rafii S, Pandolfi PP (2010) Plzf regulates germline progenitor self-renewal by opposing mTORC1. Cell 142: 468-479.

2. Seandel M, James D, Shmelkov SV, Falciatori I, Kim J, et al. (2007) Generation of functional multipotent adult stem cells from GPR125+ germline progenitors. Nature 449: 346-350.

3. Salomonis N, Schlieve CR, Pereira L, Wahlquist C, Colas A, et al. (2010) Alternative splicing regulates mouse embryonic stem cell pluripotency and differentiation. Proc Natl Acad Sci U S A 107: 10514-10519.

4. Fagoonee S, Hobbs RM, De Chiara L, Cantarella D, Piro RM, et al. (2010) Generation of functional hepatocytes from mouse germ line cell-derived pluripotent stem cells in vitro. Stem Cells Dev 19: 1183-1194.

5. Bussolati B, Moggio A, Collino F, Aghemo G, D'Armento G, et al. (2012) Hypoxia modulates the undifferentiated phenotype of human renal inner medullary CD133+ progenitors through Oct4/miR-145 balance. Am J Physiol Renal Physiol 302: F116-128.

6. Bussolati B, Bruno S, Grange C, Ferrando U, Camussi G (2008) Identification of a tumor-initiating stem cell population in human renal carcinomas. FASEB J 22: 3696-3705.

7. Rizzi R, Di Pasquale E, Portararo P, Papait R, Cattaneo P, et al. (2012) Post-natal cardiomyocytes can generate iPS cells with an enhanced capacity toward cardiomyogenic re-differentation. Cell Death Differ 19: 1162-1174.

8. Im CN, Kang NY, Ha HH, Bi X, Lee JJ, et al. (2010) A fluorescent rosamine compound selectively stains pluripotent stem cells. Angew Chem Int Ed Engl 49: 7497-7500.

9. Kang NY, Yun SW, Ha HH, Park SJ, Chang YT (2011) Embryonic and induced pluripotent stem cell staining and sorting with the live-cell fluorescence imaging probe CDy1. Nat Protoc 6: 1044-1052.
